# Targeting Transient Receptor Potential Melastatin‐2 (TRPM2) Enhances Therapeutic Efficacy of Third Generation EGFR Inhibitors against EGFR Mutant Lung Cancer

**DOI:** 10.1002/advs.202310126

**Published:** 2024-07-23

**Authors:** Zhen Chen, Karin A. Vallega, Vijay K. Boda, Zihan Quan, Dongsheng Wang, Songqing Fan, Qiming Wang, Suresh S. Ramalingam, Wei Li, Shi‐Yong Sun

**Affiliations:** ^1^ Department of Hematology and Medical Oncology Emory University School of Medicine and Winship Cancer Institute Atlanta GA 30047 USA; ^2^ Department of Pharmaceutical Sciences College of Pharmacy University of Tennessee Health Science Center Memphis TN 38163 USA; ^3^ Department of Pathology The Second Xiangya Hospital Central South University Changsha Hunan 410011 P. R. China; ^4^ Department of Internal Medicine The Affiliated Cancer Hospital of Zhengzhou University Henan Cancer Hospital Zhengzhou 450008 P. R. China

**Keywords:** apoptosis, calcium, EGFR‐TKIs, lung cancer, osimertinib, TRPM2

## Abstract

There is an urgent need to fully understand the biology of third generation EGFR‐tyrosine kinase inhibitors (EGFR‐TKIs), particularly osimertinib, and to develop mechanism‐driven strategies to manage their acquired resistance. Transient receptor potential melastatin‐2 (TRPM2) functions as an important regulator of Ca^2+^ influx, but its role in mediating therapeutic efficacies of EGFR‐TKIs and acquired resistance to EGFR‐TKIs has been rarely studied. This study has demonstrated a previously undiscovered role of suppression of TRPM2 and subsequent inhibition of Ca^2+^ influx and induction of ROS and DNA damage in mediating apoptosis induction and the therapeutic efficacy of osimertinib against EGFR mutant NSCLC. The rebound elevation represents a key mechanism accounting for the emergence of acquired resistance to osimertinib and other third generation EGFR‐TKIs. Accordingly, targeting TRPM2 is a potentially promising strategy for overcoming and preventing acquired resistance to osimertinib, warranting further study in this direction including the development of cancer therapy‐optimized TRPM2 inhibitors.

## Graphical abstract

This study reveals a previously undiscovered role of suppression of TRPM2, an important regulator of Ca^2+^ influx, in mediating therapeutic efficacy of osimertinib against EGFR mutant non‐small cell lung cancer and suggests targeting TRPM2 as a potentially promising strategy for managing acquired resistance to osimertinib and other third generation EGFR inhibitors.

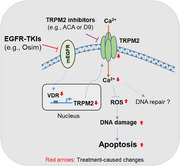

## 1 Introduction

Transient receptor potential (TRP) channels are a superfamily of ion channels involved in a large number of physiological functions including sensory functions such as taste transduction and temperature sensation, homeostatic functions like Ca^2+^ and Mg^2+^ reabsorption and osmoregulation, and cellular functions like cell motility and muscle contraction.^[^ ^1^, ^2^ ^]^ The discoveries of the fascinating roles of the TRP family of ion channels (e.g., TRPV1 and TRPM8) in mediating temperature and touch sensation won the 2021 Nobel Prize in physiology. Among this superfamily, the TRP‐melastatin (TRPM) subfamily contains eight mammalian members, TRPM1‐TRPM8, of multifunctional Ca^2+^ permeable, non‐selective cation channels with a unique C‐terminal adenosine diphosphate ribose (ADPR) pyrophosphatase domain. Aberrant TRPM2 function has been implicated in inflammatory diseases,^[^ ^3^ ^]^ several neurological disorders including ischemia/stroke, Alzheimer's disease, neuropathic pain, Parkinson's disease and bipolar disorder,^[^ ^4^ ^]^ and cancer.^[^ ^2^, ^5^ ^]^ Thus, TRPM2 has been considered a promising therapeutic target for the treatment of these diseases.^[^ ^6^ ^]^

TRPM2 is highly expressed in some types of cancer. Consistently, inhibition of TRPM2 in most studies has been connected to reduced proliferation, enhanced cell death and increased sensitivity to doxorubicin and other chemotherapeutic agents in a number of malignancies (see reviews^[^ ^2^, ^7^ ^]^), whereas a few studies have shown the opposite effects.^[^ ^8^, ^9^, ^10^, ^11^, ^12^ ^]^ The survival function of TRPM2 in cancer cells is likely associated with maintenance of mitochondrial function, cellular bioenergetics, ATP production, autophagy, reduction in cellular reactive oxygen species (ROS) levels, and DNA repair.^[^ ^2^, ^7^ ^]^ In the setting of lung cancer, TRPM2 has been seldom studied. It was reported that TRPM2 was functionally expressed in non‐small cell lung cancer (NSCLC) cells and its downregulation by knockdown significantly inhibited cell proliferation, promoted apoptosis and suppressed the growth of human lung tumor xenograft, and was tightly associated with the induction of intercellular ROS generation, increased DNA damage, and JNK activation‐induced G2/M arrest.^[^ ^13^ ^]^ Otherwise, the function of TRPM2 in lung cancer, particularly its involvement in regulating the efficacies of epidermal growth factor receptor (EGFR)‐targeted therapy and other targeted therapies against NSCLCs with different driver mutations, is largely unknown.

The discovery of EGFR activating mutations, 90% of which occur as an exon 19 deletion (19del) or exon 21 point mutation (L858R), as a predictor of patient response to EGFR tyrosine kinase inhibitors (EGFR‐TKIs) represented a milestone and paradigm shift in the treatment of NSCLC. Accordingly, targeting these mutated EGFRs with EGFR‐TKIs represents a major advance in the targeted therapy of NSCLC and the first successful targeted therapy against lung cancer. The rapid development of EGFR‐TKIs during the past two decades from the initial 1st generation (e.g., gefitinib and erlotinib) to 2nd generation (e.g., afatinib) and current 3rd generation (e.g., osimertinib; also named AZD9291 or TAGRISSO) agents as a consequence of battling against the inevitable issue of acquired resistance has substantially contributed to the improved quality of life and prolonged survival of patients with EGFR mutant (EGFRm NSCLC. Compared to early generation EGFR‐TKIs, 3rd generation EGFR‐TKIs selectively and irreversibly inhibit these mutated EGFRs and the resistant T790M mutation, which account for ≈60% of resistant cases caused by early generation EGFR‐TKIs, while sparing wild‐type (WT) EGFR. Osimertinib is now an FDA‐approved drug for patients with NSCLC relapsed to 1st generation EGFR‐TKIs due to T790M mutation and for EGFR mutation‐positive advanced NSCLC as a first‐line treatment. Unfortunately, all patients eventually develop resistance to osimertinib, resulting in disease relapse,^[^ ^14^, ^15^ ^]^ and this will inevitably be the case for other third generation EGFR‐TKIs. Acquisition of a novel resistant C797S mutation is the most common resistance mechanism particularly when osimertinib is used as a second‐line therapy. Beyond the EGFR‐dependent resistance mechanisms, there are other heterogeneous EGFR‐independent mechanisms such as *MET* or *HER2* gene amplification, acquired mutations in oncogenes (e.g., *BRAF*), and small‐cell or squamous cell transformation.^[^ ^15^, ^16^, ^17^ ^]^ However, resistance mechanisms in most cases, particularly when osimertinib is used as a first‐line therapy, are largely unknown. Hence, thorough understanding of the mechanisms of acquired resistance and the development of mechanism‐driven efficacious strategies to manage acquired resistance to osimertinib and other 3^rd^ generation EGFR‐TKIs are highly desirable and urgently needed in the clinic.

To thoroughly understand the mechanisms of acquired resistance, we believe that it is critical to fully understand the biology or action mechanisms of osimertinib in sensitive EGFRm NSCLC cells. To this end, we identified *TRPM2* as an important gene whose expression was substantially inhibited at both mRNA and protein levels in sensitive EGFRm NSCLC cells exposed to osimertinib and other EGFR‐TKIs and was elevated in several EGFRm cell lines with acquired resistance to osimertinib. Therefore, this study focused on defining the molecular mechanisms by which *TRPM2* expression is suppressed by osimertinib and its involvement in mediating therapeutic efficacy of osimertinib against EGFRm NSCLC via testing the overall hypothesis that effective inhibition of *TRPM2* expression is an essential event in maintaining long‐term therapeutic efficacy of osimertinib or other 3^rd^ generation EGFR‐TKIs in the treatment of EGFRm NSCLCs.

## 2 Results

### 2.1 Osimertinib and Other EGFR‐TKIs Inhibit TRPM2 Expression in EGFRm NSCLC Cells and Tissues

**Figure 1:**
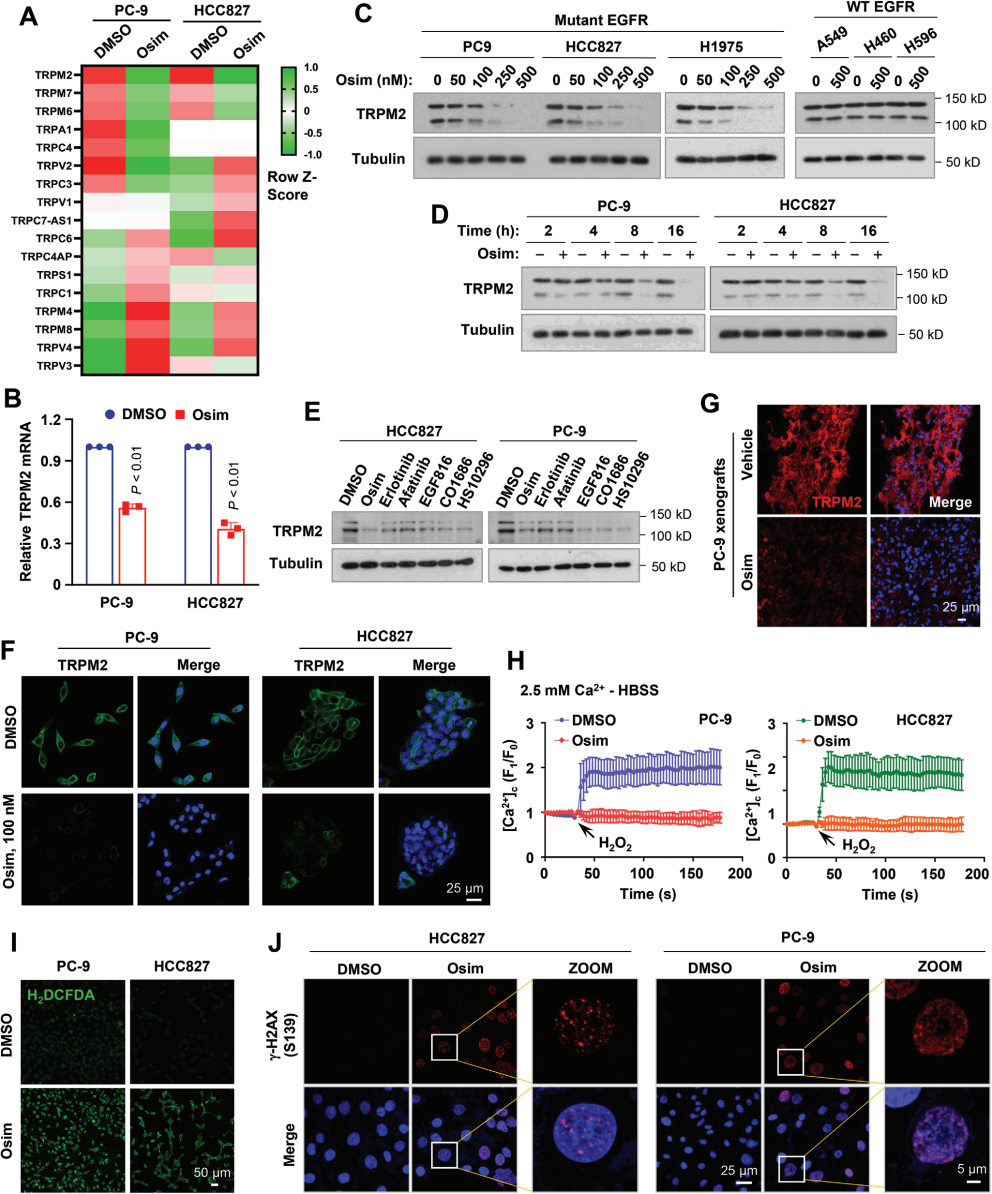
Osimertinib suppresses TRPM2 expression at both mRNA (A,B) and protein (C–G) levels accompanied with inhibition of Ca^2+^ influx (H) and induction of ROS (I) and DNA damage (J) in EGFRm NSCLC cell lines or tumors. *A*, RNA‐seq data generated from both PC‐9 and HCC827 cells exposed to DMSO or 100 nM osimertinib (Osim) for 14 h. *B*, RT‐qPCR detection of TRPM2 suppression by osimertinib in the indicated cell lines treated with DMSO or 100 nM osimertinib for 14 h. Each column is the mean ± SE of three independent experiments. Statistical differences were assessed with two‐sided unpaired Student's t‐test. *C–E*, The given cell lines were exposed to different concentrations of osimertinib as indicated for 24 h (C), 200 nM osimertinib for varied times as indicated (D), 200 nM indicated EGFR‐TKIs for 24 h (E). The proteins of interest were detected with Western blotting. *F* and *G*, TRPM2 in the indicated cell lines exposed to DMSO or 100 nM osimertinib for 24 h (F) or PC‐9 tumors treated with 15 mg kg^−1^ osimertinib for 9 days (G) was detected with IF staining. *H*, The given cell lines were exposed to DMSO or 200 nM osimertinib for 16 h and then subject to detection of Ca^2+^ influx using the Invitrogen™Fluo‐4 Direct™ Calcium Assay Kit. The cells were bathed in normal physiological saline HBSS and challenged with 3 mM H_2_O_2_ to induce a cytosolic Ca^2+^ rise. *I* and *J*, Both PC‐9 and HCC827 cells were exposed to 200 nM osimertinib for 24 h and then subject to detection of intracellular ROS generation with the H_2_DCFDA assay (I) and of γ‐H2AX foci using IF staining (J).

In our effort to understand the molecular mechanisms by which osimertinib exerts its therapeutic effect in sensitive EGFRm NSCLC cells/tumors, we identified that TRPM2 was ranked top among a few TRP genes whose expression was significantly inhibited by osimertinib in both PC‐9 and HCC827 cell lines based on analysis of our RNA sequencing (RNA‐seq) data that compared mRNA alterations between DMSO‐ and osimertinib‐treated PC‐9 or HCC827 cells^[^ ^18^ ^]^ (**Figure** **1**A). The downregulation of TRPM2 expression was confirmed by reverse transcription quantitative PCR (RT‐qPCR; Figure 1B). Analysis of TCGA data showed that TRPM2, among the TRP genes, was the only one whose expression was significantly increased in human lung adenocarcinomas (LUAD) compared with normal tissues (Figure S1A,B, Supporting Information) and its high expression was significantly associated with the poor survival of patients with EGFRm NSCLC (Figure S1C, Supporting Information). Hence, we focused our study on TRPM2. At the protein level, osimertinib at 100–500 nM range decreased TRPM2 protein levels in three EGFRm NSCLC cell lines, but did not do so even at 500 nM in 3 NSCLC cell lines with WT EGFR (Figure 1C), suggesting a mutation‐selective effect. TRPM2 reduction in osimertinib‐treated cells occurred early at 4 h and was maintained at up to 16 h (Figure 1D), indicating a rapid and sustained effect. Beyond osimertinib, other EGFR‐TKIs including erlotinib (1st generation), afatinib (2nd generation), EGF816, CO1686, and HS‐10296 (3rd generation) also decreased TRPM2 protein levels (Figure 1E). The predominant two TRPM2 bands were between 150 kD and 100 kD, as expected for this antibody. This is largely due to the presence of physiologically truncated splicing variants.^[^ ^4^, ^7^ ^]^ The specificities of these bands were also demonstrated in the subsequent TRPM2 knockdown experiments. Using immunofluorescence (IF) staining, we detected reduced cell membrane TRPM2 expression in EGFRm NSCLC cells exposed to osimertinib (Figure 1F) and also decreased TRPM2 expression in PC‐9 xenografted tissue exposed to osimertinib (Figure 1G). Collectively, it is clear that osimertinib and other EGFR‐TKIs inhibit *TRPM2* expression in EGFRm NSCLC cells.

### 2.2 Osimertinib Effectively Inhibits Ca^2+^ Influx in EGFRm NSCLC Cells and Promotes ROS Generation and DNA Damage, Critical Events in Mediating the Therapeutic Efficacy of Osimertinib

Considering that TRPM2 primarily functions as an important regulator of Ca^2+^ influx, we then determined whether downregulation of *TRPM2* expression by osimertinib causes suppression of Ca^2+^ influx in EGFRm NSCLC cells. To this end, both PC‐9 and HCC827 cell lines were treated with DMSO or osimertinib for 16 h and the effect on Ca^2+^ influx was examined. Cells were bathed in normal physiological saline HBSS, then challenged with 3 mM H_2_O_2,_ which induced a rapid cytosolic Ca^2+^ rise in both cell lines exposed to DMSO, but not in those exposed to osimertinib (Figure 1H). Hence, it is clear that osimertinib effectively inhibits Ca^2+^ influx in both PC‐9 and HCC827 cells, consistent with the effect of osimertinib on the suppression of *TRPM2* expression. In cancer cells including lung cancer cells, TRPM2 has been linked to supporting cell survival by negative regulation of ROS production and maintenance of DNA repair function^[^ ^2^, ^3^, ^13^, ^19^ ^]^ and accordingly, inhibition of TRPM2 enhanced ROS production,^[^ ^20^, ^21^ ^]^ DNA damage^[^ ^13^, ^22^, ^23^ ^]^ and apoptosis.^[^ ^13^, ^22^, ^23^ ^]^ Therefore, we further determined the effects of osimertinib on the induction of ROS production and DNA damage in EGFRm NSCLC cells. Using H_2_DCFDA and γ‐H2AX foci formation assays, we detected increased ROS generation (Figure 1I) and DNA damage (Figure 1J) in both PC‐9 and HCC827 cells exposed to osimertinib in comparison with DMSO control cells. Thus, the data clearly suggest that osimertinib induces ROS generation and DNA damage in EGFRm NSCLC cells.

To determine the involvement of ROS generation in osimertinib‐induced DNA damage and apoptosis, we applied N‐acetyl cysteine (NAC), a well‐known antioxidant, in the tested systems to see whether the presence of NAC could protect cells from induction of DNA damage and apoptosis. Indeed, osimertinib effectively induced r‐H2AX‐positive cells and apoptosis in the absence of NAC, but these effects were significantly attenuated in the presence of NAC in both PC‐9 and HCC827 cells (Figure S2, Supporting Information), suggesting that NAC protects cells from induction of DNA damage and apoptosis by osimertinib. Thus, ROS production and DNA damage are both critical events contributing to the therapeutic efficacy of osimertinib.

### 2.3 Osimertinib Downregulates *TRPM2* Expression through Suppressing Vitamin D Receptor (VDR)‐Mediated Gene Transcription in EGFRm NSCLC Cells

**Figure 2:**
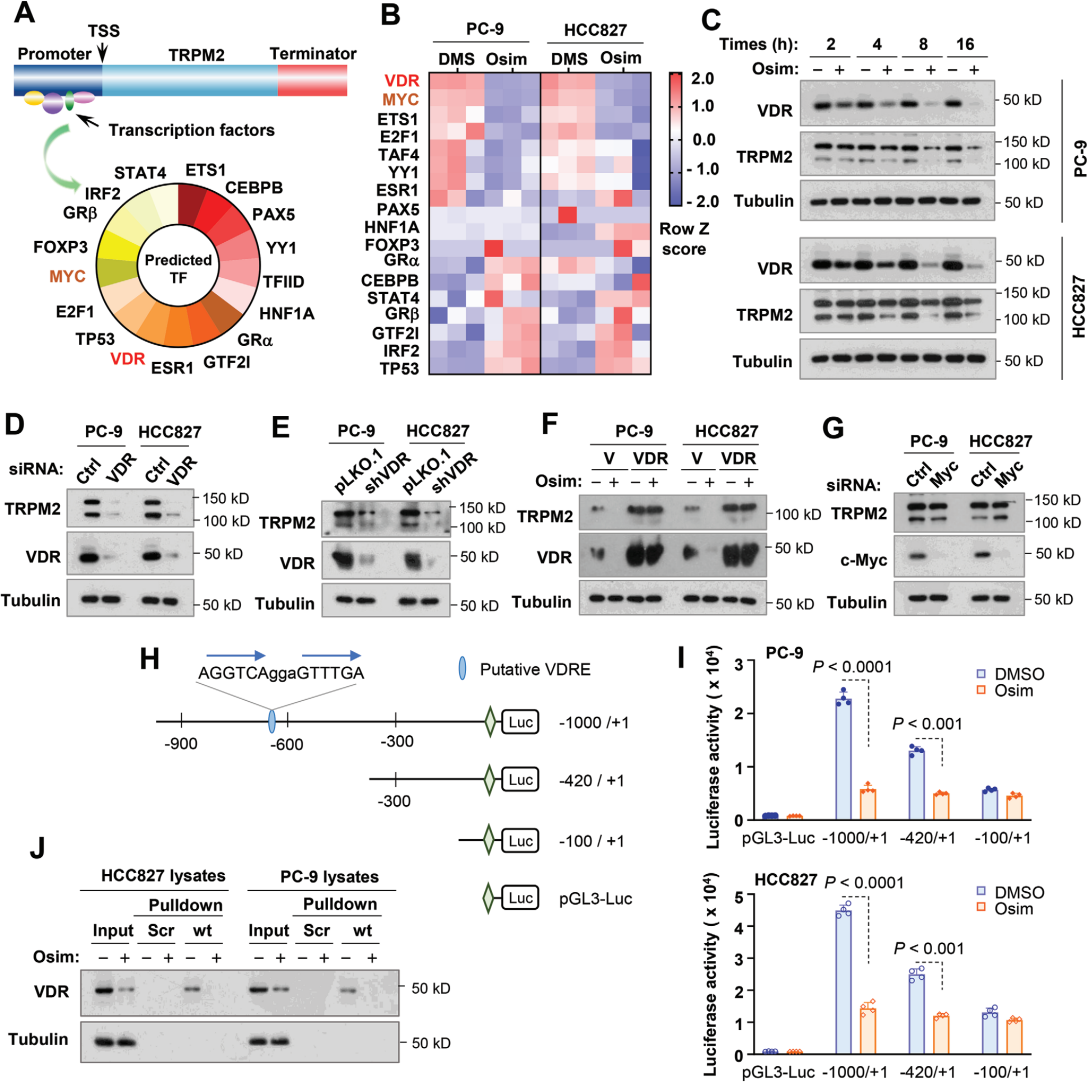
VDR is a predicted putative transcriptional factor within the TRPM2 5′‐flanking regulatory region (A), ranks top among genes suppressed by osimertinib in RNA‐seq analysis (B) and mediates TRPM2 downregulation by osimertinib in EGFRm NSCLC cells (C–F) via a VDRE present in the promoter region of *TRPM2* (H–J) independent of c‐Myc (G). *A*, Putative transcriptional factors were predicted with PROMO program. *B*, Alterations in transcriptional factors in RNA‐seq data generated from both PC‐9 and HCC827 cells exposed to DMSO or 100 nM osimertinib (Osim) for 14 h are presented in the heatmap. *C*, Both PC‐9 and HCC827 cells were exposed to DMSO or 100 nM for the indicated times. *D* and *G*, The tested cell lines were transfected with the indicated siRNAs for 48 h. *E*, The indicated cell lines were infected with lentiviruses carrying VDR shRNA followed by puromycin selection. *F*, The indicated cell lines expressing vector (V) and *VDR* gene, respectively, were exposed to DMSO or 200 nM osimertinib for 16 h. After the aforementioned treatments, the proteins of interest were detected with Western blotting. *H*, Reporter constructs harboring the TRPM2 promoter region and deleted regions. *I*, The indicated cell lines were transfected with the given reporter constructs for 24 h followed with 200 nM osimertinib for another 16 h. Cells were then harvested for luciferase assay. The data are the means ± SD of four replicate determinations. Statistical differences were assessed with two‐sided unpaired Student's *t*‐test. *J*, The indicated biotin‐oligos were incubated from whole‐cell protein lysates prepared from the indicated cell lines treated with DMSO or 200 nM osimertinib for 16 h. Pulldown assay was conducted with the streptavidin‐agarose beads followed by Western blotting to detect VDR bound to the oligos. Scr, scramble oligo; wt, VDRE‐wt oligo.

To date, there are few studies on the mechanistic regulation of *TRPM2* at the transcriptional level. We searched for possible binding sites of transcriptional factors within the *TRPM2* 5′‐flanking region containing the promoter (**Figure** **2**A) and then looked at expression of these transcription factor genes in EGFRm NSCLC cell lines exposed to osimertinib in our RNA‐seq data (Figure 2B). Based on the criterion of *P* < 0.05 and fold change (FC) +/‐ 2, the *VDR* gene was the most significantly downregulated by osimertinib in both HCC827 and PC‐9 cell lines (Figure 2B). Time‐course analysis indicated that osimertinib decreased VDR protein levels even at 2 h, which was ahead of TRPM2 reduction that occurred at 4 h post osimertinib treatment (Figure 2C). Using IF, we detected clear membrane staining of TRPM2 and cytoplasmic/nuclear staining of VDR in both PC‐9 and HCC827 cells, both of which were reduced in cells exposed to osimertinib (Figure S3, Supporting Information). Critically, knockdown of VDR with both small‐interfering RNA (siRNA) and short‐hairpin RNA (shRNA) in both PC‐9 and HCC827 cells caused TRPM2 reduction (Figure 2D,E), whereas enforced overexpression of ectopic *VDR* in these cell lines rescued TRPM2 reduction caused by osimertinib (Figure 2F). These results together robustly suggest the critical role of VDR in mediating downregulation of *TRPM2* expression by osimertinib in EGFRm NSCLC cells. This is further supported by the finding of a significant positive correlation between VDR and TRPM2 expression in EGFRm LUAD based on TCGA data analysis (Figure S4A, Supporting Information). Moreover, high VDR expression was significantly associated with worse prognosis of EGFRm NSCLC patients (Figure S4B, Supporting Information), in a similar fashion to TRPM2.

c‐Myc was also a potential transcriptional factor that regulates TRPM2 expression (Figure 2A). Although osimertinib had a limited effect on decreasing *c‐Myc* mRNA levels (Figure 2B), it substantially decreased c‐Myc protein levels in EGFRm NSCLC cells and tumors largely via facilitating c‐Myc protein degradation as we previously reported.^[^ ^18^ ^]^ However, c‐Myc knockdown in EGFRm NSCLC cells failed to result in TRPM2 reduction (Figure 2G), suggesting a nonessential role of c‐Myc in the regulation of TRPM2 expression at least in EGFRm NSCLC cells.

To demonstrate the direct regulation of TRPM2 by VDR, we identified a putative DR3 (direct repeats spaced by 3 nucleotides)‐type vitamin D response element (VDRE) located at −671/−657 (5′‐AGGTCAggaGTTTGA‐3′) in the 5′‐flanking region of the *TRPR2* gene. We thus cloned the 5′‐flanking region of the TRPM2 gene into pGL3‐Luc reporter construct with varied lengths with and without the putative VDRE as shown in Figure 2H. Co‐transfection of VDR with these reporter constructs, respectively, into HEK293 cells showed a substantial increase in luciferase activity (by 201%) in the cells transfected with pGL3‐(−1000/+1)‐Luc, but limited increase (by 68%) in cells transfected with pGL3‐(−420/+1)‐Luc and no increase in cells transfected with pGL3‐(−100/+1)‐Luc (Figure S5A, Supporting Information). Similar results were also generated in cells transfected with these reporter constructs followed by treatment with calcitriol, the active form of vitamin D that works through binding to VDR (Figure S5B, Supporting Information). Moreover, we conducted a streptavidin/biotin oligonucleotide pulldown assay to demonstrate whether VDR indeed binds to this putative VDRE (Figure S5C, Supporting Information). Clear binding of VDR to the oligonucleotide having the WT VDRE (VDRE‐wt) was detected when it was incubated with protein lysates from cells transfected with a vector plasmid (baseline binding); this binding was strongly increased when it was incubated with protein lysates from cells transfected with a VDR expression plasmid. However, binding was substantially reduced when we used an oligonucleotide harboring mutated VDRE (VDRE‐m; Figure S5D,E, Supporting Information). Hence, it is clear that this VDRE is functional. Collectively, these data strongly suggest that VDR indeed directly regulates TRPM2 expression via the VDRE (−671/−657).

When these reporter constructs were transfected into PC‐9 or HCC827 cells, the highest luciferase activities were detected in cells transfected with pGL3‐(−1000/+1)‐Luc in comparison with those transfected with other reporter constructs. When treated with osimertinib, luciferase activities were reduced the most in cells transected with pGL3‐(−1000/+1)‐Luc, and also decreased in cells transfected with pGL3‐(−420/+1)‐Luc (Figure 2I). In the oligonucleotide pulldown assay, we detected much lower amounts of VDR bound to the VDRE‐wt oligonucleotide in cell lysates from both PC‐9 and HCC827 cell lines treated with osimertinib than in cells exposed to DMSO (Figure 2J), indicating that osimertinib decreases VDR binding to the VDRE. Collectively, these results demonstrate that inhibition of VDR‐dependent gene transactivation plays a dominant role in mediating suppression of TRPM2 expression induced by osimertinib in EGFRm NSCLC cells.

### 2.4 TRMP2 Inhibition is Involved in Mediating Therapeutic Efficacy of Osimertinib against EGFRm NSCLC Cells and Tumors

**Figure 3:**
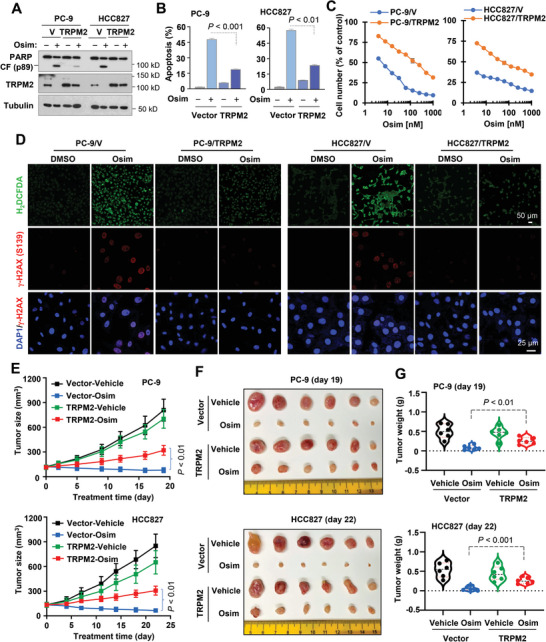
Enforced expression of ectopic *TRPM2* gene in EGFRm NSCLC cell lines attenuates the ability of osimertinib to induce apoptosis (A,B), decrease cell survival (C), promote ROS generation (D), cause DNA damage (D) and inhibit tumor growth (E–G). *A*‐*D*, The indicated cell lines expressing vector (V) or *TRPM2* gene were exposed to DMSO or 200 nM osimertinib for 24 h (A), 48 h (B), 72 h (C), or 16 h (D). The proteins of interest were detected with Western blotting (A). Annexin V‐positive cells were determined with flow cytometry (B). Cell numbers were estimated with the SRB assay (C). ROS generation and DNA damage were detected with H_2_DCFDA and γ‐H2AX foci assays, respectively. The data are means ± SDs of triplicate (B) or four replicate determinations (C). CF, cleaved form. *E*‐*G*, Mice inoculated with the indicated cell lines were treated with vehicle or osimertinib (5 mg kg^−1^, og, daily). Tumor sizes were measured at the indicated times (E) and photographed at the end of the treatments (F). Tumor weights were recorded at the end of the treatment (G). The data are means ± SEs (n = 6). Statistical differences were assessed with two‐sided unpaired Student's t‐test.

We next determined whether TRPM2 downregulation is an important event involved in mediating therapeutic efficacy of osimertinib in the treatment of EGFRm NSCLC. To this end, we enforced expression of ectopic TRPM2 in PC‐9 and HCC827 cell lines and then examined their responses to osimertinib. Compared to the vector control cells that were sensitive to osimertinib, the effects of osimertinib on inducing PARP cleavage (**Figure** **3**A), increasing annexin V‐positive cells (Figure 3B) and decreasing cell survival (Figure 3C) were substantially compromised in both PC‐9/TRPM2 and HCC827/TRPM2 cell lines. Similarly, the effects of osimertinib on induction of ROS production and DNA damage were compromised in PC‐9/TRPM2 and HCC827/TRPM2 cell lines (Figure 3D). Consistently, the effects of osimertinib on suppressing the growth of both PC‐9 and HCC827 tumors were attenuated since both PC‐9/TRPM2 and HCC827/TRPM2 tumors were significantly less responsive than their corresponding vector control tumors to osimertinib, evaluated by measuring both tumor sizes (Figure 3E,F) and weights (Figure 3G). Osimertinib also had compromised effects in decreasing Ki67‐positive and increasing cleaved PARP (cPARP)‐positive cells in both PC‐9/TRPM2 and HCC827/TRPM2 tumors compared to those in their corresponding vector control tumors (Figure S6, Supporting Information). These in vitro and in vivo results clearly demonstrate that blockade of TRPM2 downregulation attenuates the ability of osimertinib to decrease survival and induce apoptosis of EGFRm NSCLC cells and inhibit the growth of EGFRm NSCLC tumors, implying an essential role of TRPM2 downregulation in mediating the therapeutic efficacy of osimertinib against EGFRm NSCLC cells.

### 2.5 TRPM2 Expression is Elevated in EGFRm NSCLC Cell Lines with Acquired Resistance to Osimertinib and in Most EGFRm NSCLC Tissues Relapsed from EGFR‐TKI Treatment

**Figure 4:**
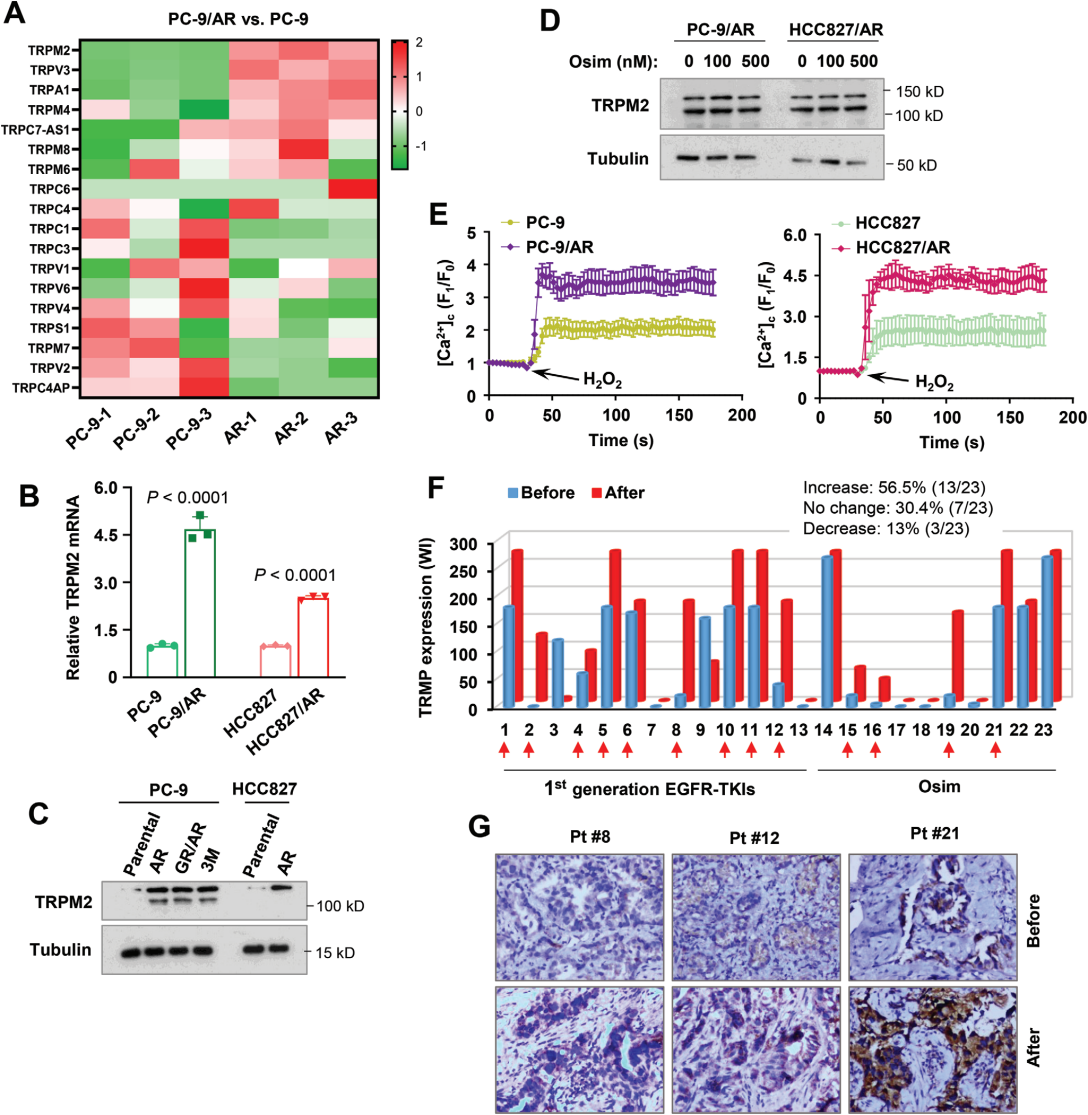
*TRPM2* expression is elevated at both mRNA (A,B) and protein (C,D) levels in EGFRm NSCLC cell lines with osimertinib acquired resistance, which exhibit increased Ca^2+^ influx (E), and in the majority of EGFRm NSCLC tissues after relapse from EGFR‐TKI treatment (F,G) *A*, Heatmap for the expression of TRPM2 and other‐related genes from RNA‐seq data between PC‐9 and PC‐9/AR cells. *B*, RT‐qPCR detection of *TRPM2* mRNA levels in the indicated cell lines. The data are means ± SDs of four replicate determinations. Statistical differences were assessed with two‐sided unpaired Student's t‐test. *C and D*, Western blotting detection of TRPM2 protein in the indicated cell lines with or without exposure to different concentrations of osimertinib (Osim) as indicated for 16 h. *E*, Detection of Ca^2+^ influx in the indicated cell lines using the Invitrogen™Fluo‐4 Direct™ Calcium Assay Kit. The cells were bathed in normal physiological saline HBSS and challenged with 3 mM H_2_O_2_ to induce a cytosolic Ca^2+^ rise. *F* and *G*, TRPM2 in human EGFRm NSCLC issues were detected with IHC (F) and the representative IHC pictures are shown (G).

Following the above findings, we then compared basal levels of *TRPM2* expression between EGFRm NSCLC cell lines and their derived cell lines with acquired resistance to osimertinib or other EGFR‐TKIs. We found that *TRPM2* expression was significantly elevated in PC‐9/AR cells (PC‐9 cells with acquired resistance to osimertinib) compared with that in PC‐9 cells in our RNA‐seq analysis (**Figure** **4**A). This finding was confirmed with RT‐qPCR in both PC‐9/AR and HCC827/AR cells (Figure 4B). The elevated basal levels of TRPM2 protein in different osimertinib‐resistant cell lines were also detected by Western blotting (Figure 4C). Moreover, osimertinib lost its ability to decrease TRPM2 protein levels in the tested osimertinib‐resistant NSCLC cell lines as detected with Western blotting (Figure 4D) and IF (Figure S7, Supporting Information). Consistent with the elevation of TRPM2, both PC‐9/AR and HCC827/AR cells possessed higher capacity for Ca^2+^ influx than their corresponding parent cell lines (Figure 4E), implying increased intracellular levels of Ca^2+^ in these osimertinib‐resistant cells.

In human EGFRm NSCLC tissues relapsed from treatment with EGFR‐TKIs including osimertinib, TRPM2 elevation was detected in 56.5% (13/23) of cases compared with their matched pre‐treatment tissues, whereas TRPM2 expression remained unchanged in 30.4% (7/23) of cases post relapse (Figure 4F,G). Thus, TRPM2 elevation occurs not only in EGFRm NSCLC cell lines with acquired resistance to osimertinib, but also in the majority of EGFRm NSCLC tissues relapsed from EGFR‐TKI treatment.

### 2.6 Genetic Knockdown of *TRPM2* Expression in Osimertinib‐Resistant Cell Lines Sensitizes the Cells to Osimertinib Both In vitro and In vivo

**Figure 5:**
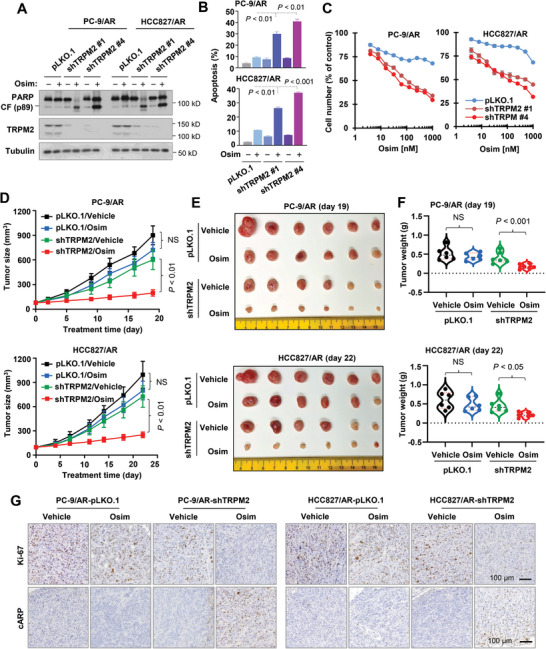
Genetic suppression of *TRPM2* expression via gene knockdown in osimertinib‐resistant cells restores their responses to osimertinib in inducing apoptosis (A,B), decreasing cell survival (C) and enhancing suppression of tumor growth (D–F) with augmented effects on decreasing Ki‐67 and inducing PARP cleavage (G) in vivo. *A*‐*C*, The indicated cell lines expressing pLKO.1 or shTRPM2 were exposed to DMSO or 200 nM osimertinib (Osim) for 24 h (A), 48 h (B) or 72 h (C). The proteins of interest were detected with Western blotting (A). Annexin V‐positive cells were determined with flow cytometry (B). Cell numbers were estimated with the SRB assay (C). The data are means ± SDs of triplicate (B) or four replicate (C) determinations. CF, cleaved form. *D*‐*F*, Mice inoculated with the indicated cell lines were treated with vehicle or osimertinib (5 mg kg^−1^, og, daily). Tumor sizes were measured at the indicated times (D) and photographed at the end of the treatments (E). Tumor weights were recorded at the end of the treatment as well (F). The data are means ± SEs (n = 6). *G*, Ki‐67 and cPARP in the indicated tissues were detected with IHC. NS, not significant. Statistical differences were assessed with two‐sided unpaired Student's t‐test.

To determine the possible involvement of TRPM2 elevation in mediating cell resistance to osimertinib, we used both siRNA and shRNA strategies to knock down *TRPM2* expression in osimertinib‐resistant cell lines and then assayed their responses to osimertinib. As expected, shRNA‐mediated knockdown of TRPM2 in osimertinib‐resistant cell lines including PC‐9/AR and HCC827/AR (**Figure** **5**A) rendered the cells more sensitive to osimertinib than their corresponding control resistant cells in terms of apoptosis induction as evaluated by detection of both PARP cleavage (Figure 5A) and annexin V‐positive cells (Figure 5B) and cell survival decrease (Figure 5C). Similarly, TRPM2 knockdown with a siRNA also sensitized these cell lines to undergo apoptosis induced by osimertinib, as evidenced by enhanced PARP cleavage (Figure S8A, Supporting Information). Moreover, while osimertinib induced limited ROS production and γ‐H2AX foci formation in PC‐9/AR and HCC827/AR cells transfected with a control siRNA, osimertinib substantially increased both ROS production and γ‐H2AX foci formation in these cell lines transfected with a TRPM2 siRNA (Figure S8B, Supporting Information), suggesting that genetic knockdown of TRPM2 in osimertinib‐resistant cells restores the ability of osimertinib to induce ROS generation and DNA damage. In agreement, both PC‐9/AR/shRNA and HCC827/AR/shRNA tumors were significantly responsive to osimertinib, whereas their corresponding control tumors minimally responded to osimertinib as evaluated by measuring both tumor sizes (Figure 5D,E) and weights (Figure 5F). In agreement, we detected substantially reduced cells with Ki67 expression and increased cells positive for cPARP in both PC‐9/AR/shRNA and HCC827/AR/shRNA tumors treated with osimertinib, but not in their corresponding control tumor tissues exposed to osimertinib (Figure 5G), indicating an impact of TRPM2 knockdown on increasing cell sensitivity to osimertinib‐induced proliferation inhibition and apoptosis. Taking these in vitro and in vivo data together, it is clear that enforced suppression of TRPM2 expression with gene knockdown in osimertinib‐resistant cell lines sensitizes the cells to osimertinib, further suggesting a critical role of TRPM2 modulation in mediating EGFRm NSCLC cell responses to osimertinib.

### 2.7 Chemical Inhibition of TRPM2 Synergizes with Osimertinib in Inducing Apoptosis and Decreasing the Survival of Osimertinib‐Resistant Cells and Inhibiting the Growth of Osimertinib‐Resistant Tumors

**Figure 6:**
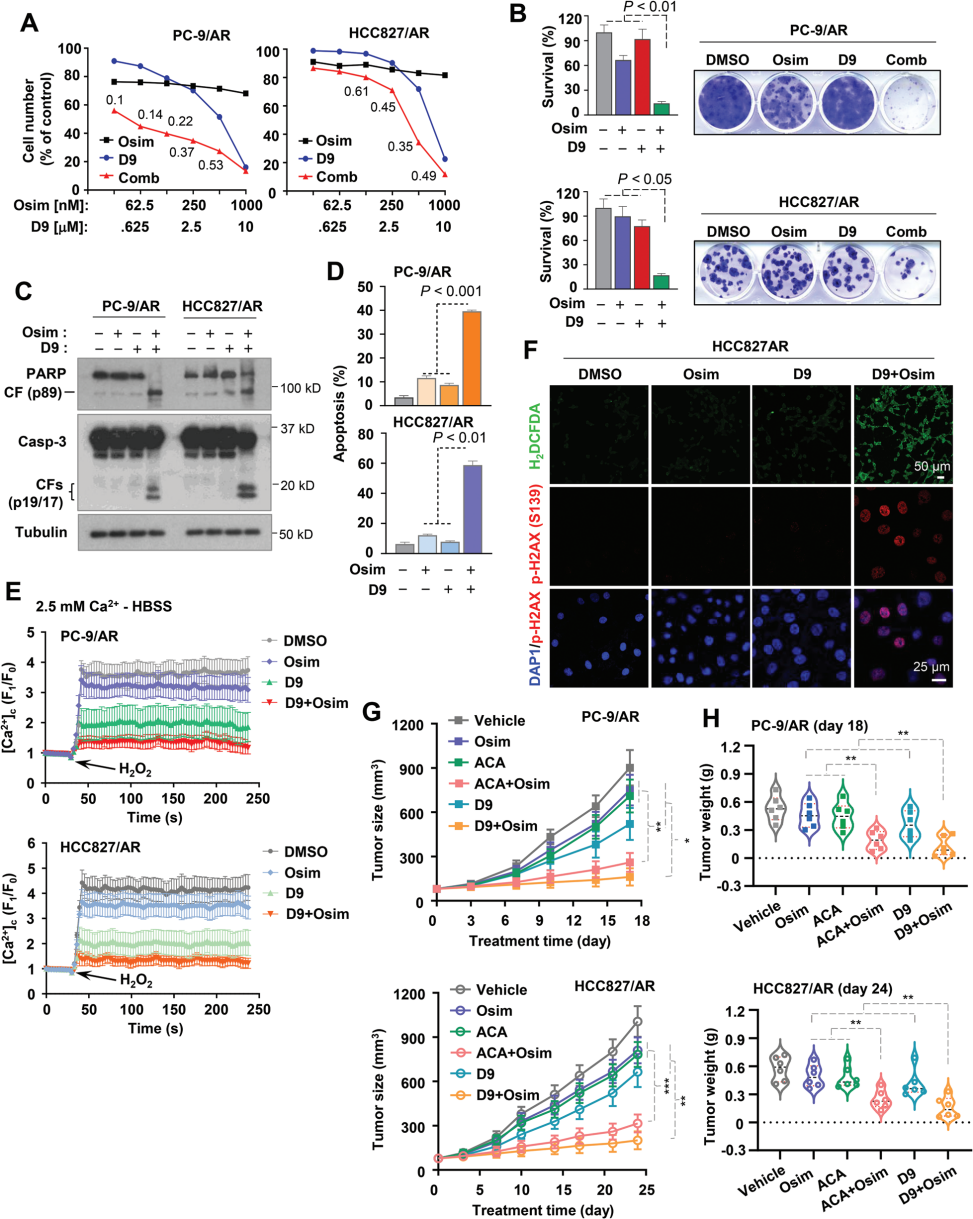
D9 in combination with osimertinib synergistically decreases cell survival (A), inhibits colony formation and growth (B), induces apoptosis (C,D), reduces Ca^2+^ influx (E), and induces ROS production and DNA damage (F) in osimertinib‐resistant EGFRm NSCLC cell lines and augments the growth inhibition of osimertinib‐resistant tumors in vivo, as does the combination of ACA and osimertinib (G,H). *A*, The given cell lines were treated with varied concentrations of the tested agents either alone or in combinations for 3 days. Cell numbers were then measured by the SRB assay (A) and CIs were then calculated and presented inside the graphs. The data are means ± SDs of four replicate determinations. *B*, The tested cell lines seeded in 12‐well plates were treated with 50 nM osimertinib, 250 nM D9, or their combination, which were repeated with fresh medium every 3 days. After 10 days, the cells were fixed, stained with crystal violet dye, imaged, and counted. Columns are means ± SDs of triplicate determinations. *C* and *D*, The tested cell lines were exposed to 200 nM osimertinib, 2.5 µM D9, or their combination for 36 h (C) or 48 h (D). The proteins of interest were detected with Western blotting (C) and apoptotic cells were detected with annexin V staining/flow cytometry (D). Each column represents mean ± SD of triplicate treatments. *E*, The indicated cell lines exposed to DMSO, 100 nM osimertinib, 2.5 µM D9, or the combination of osimertinib and D9 for 36 h were bathed in normal physiological saline HBSS and challenged with 2.5 mM H_2_O_2_ to induce a cytosolic Ca^2+^ rise followed with the detection of Ca^2+^ influx using the Invitrogen™Fluo‐4 Direct™ Calcium Assay Kit. *F*, HCC827/AR cells were exposed to DMSO, 100 nM osimertinib, 2.5 µM D9, or the combination of osimertinib and D9 for 36 h and then assayed for ROS generation with H_2_DCFDA dye and for p‐H2AX foci formation by p‐H2AX staining. *G* and *H*, Mice inoculated with the indicated cell lines were treated with vehicle, osimertinib (5 mg kg^−1^, og, daily), D9 (25 mg kg^−1^, ip, daily), or the combination of osimertinib and D9. Tumor sizes were measured at the indicated times (G) and photographed at the end of the treatments. Tumor weights were recorded at the end of the treatment (H). The data are means ± SEs (n = 6). *, P < 0.05; **, P < 0.01; ***, P < 0.001. Statistical differences were evaluated with one‐way ANOVA test.

To potentially translate our findings to a realistic strategy in the clinic for the treatment of EGFRm NSCLC relapsed from osimertinib treatment, we further screened several small molecule TRPM2 inhibitors or inhibitors with TRPM2‐inhibitory activity^[^ ^6^ ^]^ including N‐(p‐amylcinnamoyl)anthranilic acid (ACA),^[^ ^6^, ^24^ ^]^ JNJ‐28583113,^[^ ^25^ ^]^ ZA‐10, ZA‐18,^[^ ^26^ ^]^ D9,^[^ ^27^ ^]^ econazole, clotrimazole, 2‐APB, FFA, 12‐deacetylscalaradial and carvacrol for their abilities to synergize with osimertinib in decreasing the survival of osimertinib‐resistant cells. We found that ACA, ZA‐10, ZA‐18, JNJ‐28583113, and particularly D9, when combined with osimertinib, were significantly more active than each agent alone in killing both PC‐9/AR and HCC827/AR cells (Figure S9A, Supporting Information). The combination of osimertinib with each of these agents had combination indexes (CIs) < 1, indicating synergistic effects on decreasing the survival of both HCC827/AR and PC‐9/AR cell lines (**Figure** **6**A; Figure S9B, Supporting Information). The combination of D9 with HS‐10296, another third generation EGFR‐TKI, was also more effective than either agent alone in decreasing the survival of both PC‐9/HSR (resistant to HS‐10296) and HCC827/HSR cells with CIs of < 1 (Figure S10, Supporting Information). In the subsequent experiments, we primarily used D9 as well as ACA in some experiments. Using a colony formation assay that allows us to treat the cells repeatedly for a relatively long time, we further showed that the combination of D9 and osimertinib effectively inhibited the formation and growth of both PC‐9/AR and HCC827/AR colonies, whereas both agents alone had minimal or no effect (Figure 6B). Similarly, the combination of osimertinib and D9 was significantly more effective than either agent alone in inducing cleavage of both caspase‐3 and PARP and increasing annexin V‐positive cells in both PC‐9/AR and HCC827/AR cells (Figure 6C,D), indicating enhanced induction of apoptosis. Moreover, we further determined whether the combination enhances suppression of Ca^2+^ influx and induction of ROS generation and DNA damage in these osimertinib‐resistant cell lines. As presented in Figure 6E, while osimertinib alone had limited effects on suppressing Ca^2+^ influx in both PC‐9/AR and HCC827/AR cells, D9 alone apparently inhibited Ca^2+^ influx in these cell lines. However, the combination of osimertinib and D9 exerted the most potent effects on blocking Ca^2+^ influx in both cell lines (Figure 6E), indicating enhanced suppression of Ca^2+^ influx in osimertinib‐resistant cell lines. Consistently, this combination, but not each single agent, apparently induced ROS generation and DNA damage as evidenced by increased H_2_DCFDA fluorescent signal and p‐H2AX foci formation in both PC‐9/AR (Figure S11, Supporting Information) and HCC827/AR (Figure 6F) cell lines.

Following these in vitro studies, we then validated the effects of the combination of osimertinib with a TRPM2 inhibitor on the growth of osimertinib‐resistant tumors. In both PC‐9/AR and HCC827/AR xenograft models, the combination of osimertinib combined with either D9 or ACA was significantly more effective than either single agent, which had very limited or no inhibitory effect, in inhibiting the growth of the tested xenografts as evaluated by measuring both tumor sizes (Figure 6G; Figure S12A, Supporting Information) and weights (Figure 6H). Consistently, these combinations were effective in decreasing cells positive for Ki67 staining and increasing cells positive for cPARP staining in both PC‐9/AR and HCC827/AR tumor tissues while either agent in the combination alone had minimal or no effect (Figure S12B, Supporting Information), indicating enhanced inhibition of cell proliferation with induction of apoptosis in vivo. The body weights of mice receiving either combination treatment were basically comparable to these of mice in other treatment groups (Figure S12C, Supporting Information), indicating that the combinations are well‐tolerated in mice.

### 2.8 The Combination of D9 and Osimertinib Effectively Enhances Therapeutic Efficacy and Delays the Emergence of Acquired Resistance to Osimertinib

**Figure 7:**
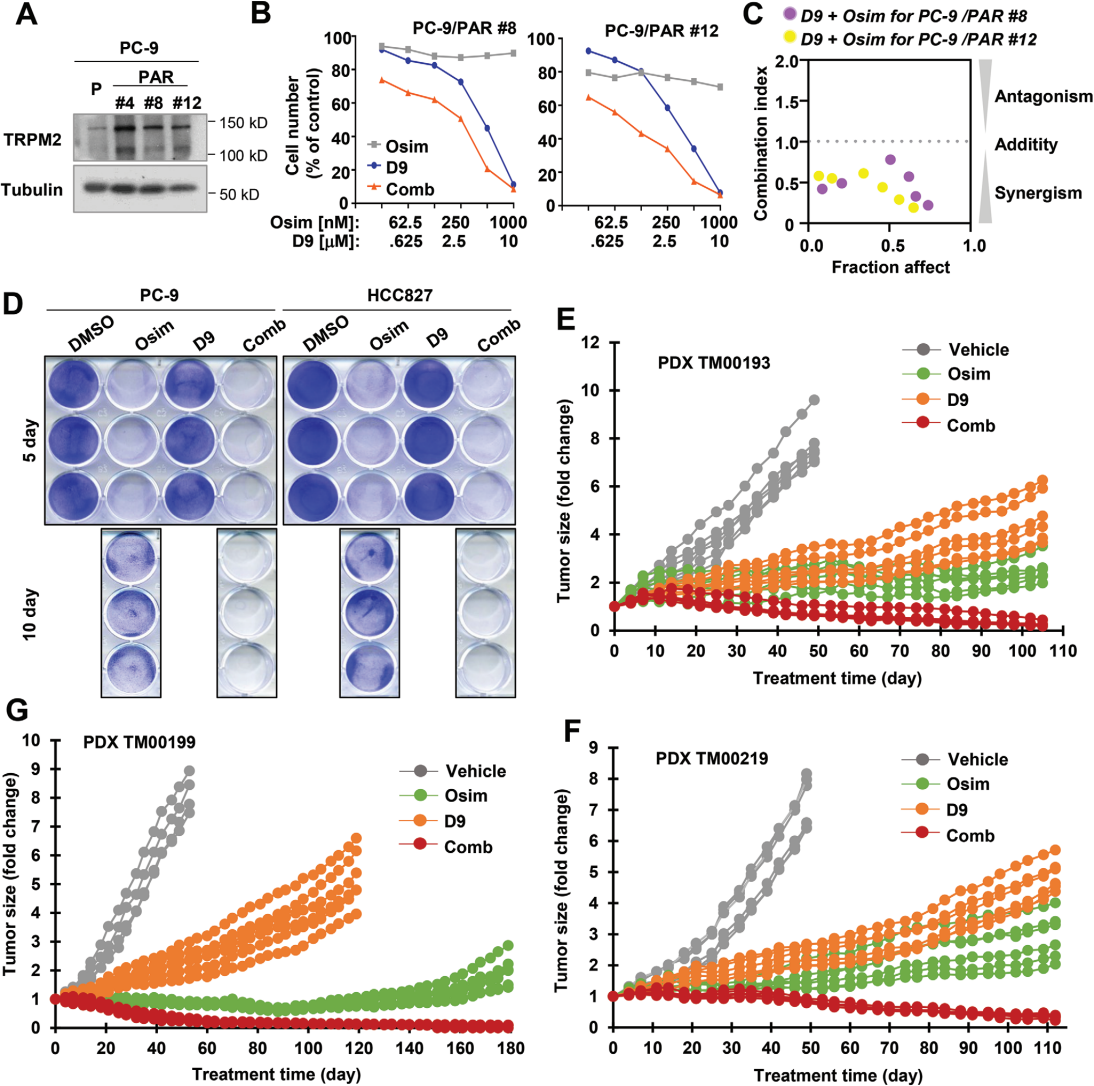
Osimertinib in combination with D9 synergistically decreases the survival of EGFRm NSCLC cell lines with primary resistance to osimertinib that possess elevated TRPM2 expression (A), eliminates DTCs (B) and regresses different EGFRm PDX tumors in vivo with long‐term remissions (D–F). *A*, Detection of TRPM2 basal levels in the indicated cell lines with Western blotting. *B and C*, The given cell lines were exposed to varied concentrations of osimertinib (Osim), D9 alone as indicated and their combination for 3 days. Cell numbers were then determined with the SRB assay and CIs were calculated (C). The data are means ± SDs of four replicate determinations. *D*, The indicated cell lines seeded in 12‐well plates were treated with 50 nM osimertinib, 250 nM D9, or their combination; these treatments were repeated with fresh medium every 2 days. After 5 or 10 days, the cells were fixed, stained with crystal violet dye and imaged. *E*‐*G*, The indicated PDXs in nude mice (6 tumors per group) were treated with vehicle, 5 mg kg^−1^ osimertinib (daily, og), 25 mg kg^−1^ D9 (daily, ip) or their combination. Tumor growth curves for each tumor are presented.

It is currently recognized that the presence of primarily resistant clones and drug tolerant persister cells (DTCs) in sensitive EGFRm NSCLC cell populations represents a primary mechanism accounting for the emergence of acquired resistance to EGFR‐TKIs.^[^ ^28^, ^29^ ^]^ We found that three cell lines with primary resistance to osimertinib, which were originally derived from PC‐9 cells,^[^ ^30^ ^]^ possessed elevated levels of TRPM2 (**Figure** **7**A). The combination of D9 and osimertinib was more active than either agent alone in decreasing the survival of these cell lines with CIs of < 1 (Figure 7B,C), showing a synergistic effect. Although initial treatment with osimertinib or in combination with D9 effectively inhibited the growth of both PC‐9 and HCC827 cells, DTCs were detected after a sustained 10‐day treatment in cells exposed to osimertinib, but not in those treated with the combination of D9 and osimertinib (Figure 7D), indicating that the combination is effective in eliminating DTCs. Following these in vitro studies, we then conducted resistance delay experiments in three different EGFRm NSCLC patient‐derived xenografts (PDXs), which were all sensitive to osimertinib albeit with varied degrees (Figure 7E–G). As the treatment with osimertinib was continued for a long period (e.g., 100 days in TM00199), these tumors did not respond to osimertinib and gradually grew larger. These PDXs also responded to D9 for the first 30 days and then resumed their growth. However, the combination of osimertinib and D9 maintained suppressive effects on the growth of these tumors, resulting in continuous tumor shrinkage starting from ≈20–40 days until the end of the experiments (100‐180 days). By the time we completed the experiments, we detected either minimal or no residual tumors (Figures 7E–G). These results robustly demonstrate the high potential of osimertinib in combination with D9 in effectively delaying or even preventing the emergence of acquired resistance to osimertinib in EGFRm NSCLC. Importantly, the body weights of mice receiving the combination treatment were comparable to those in mice receiving either vehicle or a single agent over a long treatment period (Figure S13, Supporting Information), indicating that the combination does not decrease mouse body weights. Hence, the combination of osimertinib with D9 is well tolerated.

## 3 Discussion

TRPM2 is known to be involved in diverse biological functions primarily as a cellular sensor for oxidative stress and temperature^[^ ^6^ ^]^ and has been considered a promising therapeutic target for the treatment of some diseases, particularly ischemic injury and neurologic disorders.^[^ ^4^, ^6^ ^]^ The involvement of TRPM2 in lung cancer, particularly its connection to the regulation of the efficacies of EGFR‐targeted therapy and other targeted therapies against NSCLCs with different driver mutations, is largely unknown (literature search for “TRPM2 and lung cancer” will yield many reports on clusterin, which is also named TRPM2, but is a totally different gene or protein). The current study has clearly demonstrated that osimertinib and other EGFR‐TKIs inhibit TRPM2 expression via a VDR‐dependent mechanism primarily in EGFRm NSCLC cells and tumors accompanied with suppression of Ca^2+^ influx and induction of ROS generation and DNA damage; these events are critical for osimertinib to exert its apoptosis‐inducing activity and therapeutic efficacy against the growth of EGFRm NSCLC tumors because enforced expression of ectopic TRPM2 in these cell lines compromised the effects of osimertinib on inducing ROS, DNA damage and apoptosis and on suppressing the growth of EGFRm NSCLC tumors in vivo. Moreover, we have demonstrated that rebound increase in TRPM2 expression, which is resistant to modulation by osimertinib, in EGFRm NSCLC with acquired osimertinib‐resistance and tissues relapsed from EGFR‐TKI treatment, is a critical mechanism accounting for the emergence of acquired resistance to osimertinib since genetic suppression of TRPM2 expression by gene knockdown in osimertinib‐resistant EGFRm NSCLC cells substantially sensitized the cells to generate ROS and DNA damage, undergo apoptosis and slow down growth in vivo upon osimertinib treatment. Hence, our findings convincingly reveal a previously undiscovered connection between TRPM2 modulation and response of EGFRm NSCLC cells to osimertinib and likely other EGFR‐TKIs and also suggest a novel strategy for overcoming acquired resistance to osimertinib and other third generation EGFR‐TKIs via targeting TRPM2. This new knowledge strongly supports TRPM2 as an important target for cancer therapy as well.

Beyond the specific genetic inhibition of TRPM2 that leads to sensitization of osimertinib‐resistant cells to osimertinib, we also used multiple different small molecule TRPM2 inhibitors, particularly the TRPM2‐selective inhibitor, D9,^[^ ^27^ ^]^ and generated similar results when combined with osimertinib in decreasing the survival and inducing apoptosis of osimertinib‐resistant cells, inducing ROS generation and DNA damage, and inhibiting the growth of osimertinib‐resistant tumors, demonstrating promising activities in overcoming acquired resistance to osimertinib. In addition to the strategy of overcoming acquired resistance after relapse occurs, which is a passive and late action, another active and powerful strategy is to employ an intervention action early during treatment to delay or even prevent the emergence of acquired resistance.^[^ ^29^, ^31^ ^]^ In this study, the combination of osimertinib and D9 effectively eliminated cells with primary resistance to osimertinib and DTCs, which are all origins of emergence of acquired resistance to osimertinib,^[^ ^29^ ^]^ and potentiated suppression of different EGFRm NSCLC PDXs with delayed emergence of acquired resistance to osimertinib, indicating encouraging potential in delaying the emergence of acquired resistance to osimertinib. These in vitro and in vivo data convincingly demonstrate the promising potential of targeting TRPM2 in managing, including overcoming and delaying, acquired resistance to osimertinib and likely to other third generation EGFR‐TKIs as well.

We recognize that ACA is not a pure TRPM2 inhibitor and inhibits phospholipase A2 as well.^[^ ^24^, ^32^ ^]^ However, D9,^[^ ^27^ ^]^ JNJ‐28583113,^[^ ^25^ ^]^ ZA‐10 and ZA‐18^[^ ^26^ ^]^ are relatively selective inhibitors of TRPM2. Together with TRPM2 knockdown in osimertinib‐resistant cells and tumors, we can confidently suggest targeting TRPM2 as a potential strategy for managing acquired resistance to osimertinib or to other third generation EGFR‐TKIs, warranting further study in this direction. Since targeting TRPM2 has potential in the treatment of a wide variety of CNS diseases, including ischemia/stroke, Alzheimer's disease, neuropathic pain, bipolar disorder, and Parkinson's disease, great efforts have been made to develop TRPM2 specific inhibitors for the past decades although progress has been slow.^[^ ^3^, ^4^, ^6^ ^]^ Unfortunately, these have not been specifically developed for the purpose of cancer treatment, implying that they may not be optimized for cancer treatment. Like any other small molecule drugs, there may be concerns with potential side effects (such as those related to temperature sensation and regulation) of TRPM2 inhibitors. Favorable data show that genetic deletion of TRPM2 is well tolerated and does not appear to alter behavior.^[^ ^33^ ^]^ Our long‐term treatment of mice inoculated with EGFRm NSCLC PDXs with D9 and osimertinib combination for up to 180 days was well tolerated, while exerting a promising delay in the emergence of acquired resistance to osimertinib. Collectively, efforts may be warranted to develop cancer‐optimized TRPM2 inhibitors. Moreover, TRPM2 inhibitor‐based antibody‐drug conjugations (ADCs) or targeted nanoparticle drug delivery of TRPM2 inhibitors are other options for TRPM2‐targeted cancer therapy via reducing its systemic effects.

To date, there are few studies on the mechanistic regulation of *TRPM2* expression at a transcriptional level. The finding of VDR‐mediated transcriptional regulation of TRPM2 expression is novel. VDR preferentially binds to a direct repeat of the hexameric motif (A/G)G(G/T)T(C/G)A spaced by three nucleotides, which is often referred to as a DR3 type response element.^[^ ^34^ ^]^ The functional DR3 VDRE (5′‐AGGTCAggaGTTTGA‐3′) located at (−671/−657) promoter region of *TRPM2* gene as demonstrated in this study indicates that TRPM2 is a previously undiscovered direct target gene of VDR. Hence, our novel findings add new knowledge of the molecular mechanisms by which TRPM2 expression is regulated. It is known that vitamin D/VDR signaling plays a critical role in regulating Ca^2+^ homeostasis.^[^ ^35^, ^36^ ^]^ Hence, the VDR positive regulation of TRPM2 expression is consistent with the important function of VDR. This previously unrevealed connection also increases our understanding of the biology underlying vitamin D/VDR in regulation of Ca^2+^ homeostasis. It has been shown that TRPV1 and TRPV6 are positively regulated by active vitamin D, albeit through different mechanisms: vitamin D functions as an endogenous agonist or ligand to activate TRPV1 via direct binding,^[^ ^37^, ^38^ ^]^ whereas it activates TRPV6 via VDR that directly binds to VDREs present in the TRPV6 promoter region.^[^ ^39^ ^]^ Despite both TRPM2 and TRPV6 functioning as direct target genes of VDR, the expression of TRPV6 was increased in PC‐9 cells although its expression was not detected in HCC827 cells in our RNA‐seq analysis (Figure 1A). Hence, osimertinib has a specific effect on downregulation of TRPM2 expression in EGFRm NSCLC cells. Both PI3K/Akt and MEK/ERK are well‐known downstream signaling of EGFR. Whether there is a connection between suppression of AKT and/or ERK and TRPM2 inhibition is not the focus of this study, but may deserve a further investigation in the future.

Similar to TRPM2, it has been demonstrated that the expression of both TRPV1 and TRPV6 is upregulated in various types of cancer and is associated with cancer progression and poor prognosis.^[^ ^40^, ^41^ ^]^ Therefore, efforts have been made to target these Ca^2+^ ion channels as a potential cancer therapeutic strategy.^[^ ^2^, ^40^, ^41^ ^]^ However, vitamin D and activation of VDR signaling in general exert anticancer activity including potential prevention and treatment of cancer although clinical evidence still lacks.^[^ ^36^, ^42^ ^]^ In some preclinical studies, VDR deficiency and application of active vitamin D3 were shown to reduce lung cancer incidence and metastasis. High VDR expression is associated with better prognosis of patients with lung cancer.^[^ ^43^ ^]^ However, high levels of plasma 25‐hydroxyvitamin D are associated with worse survival in lung cancer patients (particularly male patients).^[^ ^44^, ^45^ ^]^ Hence, a definitive relationship between lung cancer and vitamin D use has not been robustly established, particularly in humans. Recent works have suggested the oncogenic function of VDR^[^ ^46^, ^47^ ^]^ and targeting VDR with a VDR antagonist for effective treatment of cancer.^[^ ^48^, ^49^ ^]^ Nonetheless, further study is needed to understand the biological significance and role of vitamin D‐ or VDR‐mediated regulation of these TRP ion channels, particularly TRPM2, in different types of cancer.

The connection between VDR and TRPM2 prompts us to wonder whether modulation of VDR may also impact osimertinib's therapeutic efficacy. It is important to keep in mind that VDR, as a transcriptional factor, regulates the expression of many other genes beyond TRPM2, resulting in diversified biological functions. As a consequence, targeting VDR may lead to profound and even opposing biological events including side effects and toxicity. Moreover, activation of VDR signaling (e.g., with active vitamin D) is in general associated with suppression of tumor growth and potentiation of cancer therapy.^[^ ^42^, ^50^ ^]^ Hence, it makes sense to target regulation of TRPM2 expression, a downstream event of VDR, for more specific regulation of osimertinib's therapeutic efficacy. Nonetheless, the potential effect of osimertinib combined with VDR inhibition on the growth of EGFRm NSCLC cells may be investigated as a future direction given the fact that a VDR antagonist named MeTC7 has effective antitumor activity.^[^ ^48^, ^49^ ^]^

In this study, TRPM2 elevation was detected in close to 60% of EGFRm NSCLC tissues from patients whose disease relapsed from treatment with EGFR‐TKIs including osimertinib. This suggests that there are other resistance mechanisms accounting for the relapsed cases that did not show TRPM2 elevation. Hence, it is very likely that the combined strategy of osimertinib with a TRPM2 inhibitor will work well in these relapsed NSCLC with elevated TRPM2 because of the dependency of these tumors on TRPM2. Hence, detection of TRPM2 elevation in relapsed cases may be used as a predictive biomarker for selecting patients with disease relapse from osimertinib treatment in order to receive this therapeutic strategy for achieving possible clinical benefit. Patients with relapsed NSCLC without TRPM2 elevation should seek alternative strategies for treatment better based on the underlying resistance mechanisms.

Osimertinib was reported to promote ROS generation, contributing to its induction of apoptosis and autophagy.^[^ ^51^, ^52^ ^]^ However, the underlying mechanism is unknown. Under the context of TRPM2 inhibition, particularly in cancer cells, Ca^2+^ influx into the cell and into mitochondria is reduced, leading to decreased expression of some key transcription factors such as HIF‐1/2α, and Nrf2 that will result in suppression of antioxidant enzymes such as SOD and subsequent induction of ROS generation.^[^ ^2^ ^]^ In our study, osimertinib apparently enhanced ROS generation in different EGFRm NSCLC sensitive to osimertinib; this event contributes to DNA damage and apoptosis induced by osimertinib since the application of NAC substantially attenuated the ability of osimertinib to enhance DNA damage and induce apoptosis. Enforced expression of ectopic TRPM2 in sensitive EGFRm NSCLC cells abrogated the ability of osimertinib to induce ROS generation, DNA damage, and apoptosis, whereas genetic inhibition of TRPM2 using gene knockdown in osimertinib‐resistant cell lines restored the effect of osimertinib on induction of ROS generation, DNA damage, and apoptosis. Hence, it is apparent that ROS generation is secondary to inhibition of TRPM2 and subsequent Ca^2+^ influx. Thus, our findings in this study provide mechanistic insight into the induction of ROS generation by osimertinib.

In summary, the current study has demonstrated that suppression of TRPM2 followed by inhibition of Ca^2+^ influx and induction of ROS and DNA damage is a critical event in mediating the induction of apoptosis and the therapeutic efficacy of osimertinib against EGFRm NSCLC. The rebound elevation of TRPM2 represents a key mechanism accounting for the emergence of acquired resistance to osimertinib and likely to other third generation EGFR‐TKIs. Accordingly, targeting TRPM2 is a potentially promising strategy for managing, including overcoming and delaying, acquired resistance to osimertinib. Our findings support TRPM2 as a potential cancer therapeutic target and warrant further study in this direction including the development of cancer therapy‐optimized TRPM2 inhibitors.

## 4 Experimental Section

### Reagents

ACA, JNJ‐28583113, econazole, clotrimazole, 2‐APB, carvacrol, flufenamic acid, and NAC were purchased from MedChemExpress (MCE; Monmouth Junction, NJ). The compounds D9, ZA‐10, ZA‐18, and 12‐deacetylscalaradial were synthesized in the Li lab following the reported synthetic routes.^[^ ^26^, ^27^ ^]^ TRPM2 antibody (ACC‐043) was purchased from Alomone labs (Jerusalem, Israel). Vitamin D receptor (VDR; #12 550), c‐Myc (#5605), and cPARP (#5625) antibodies were purchased from Cell Signaling Technology, Inc (Beverly, MA). Anti‐phospho‐histone H2AX (Ser139; γ‐H2AX) antibody was purchased from MilliporeSigma (Cat # 05–636; St. Louis, MO). Ki‐67 antibody (MA5‐14520), H2DCFDA (D399), Fluo‐4 (F14201), DAPI (# 62 248), secondary antibodies of Alexa Fluor 488‐Donkey anti‐mouse (A32766) and Alexa Fluor 568‐Donkey anti‐rabbit (A10042) were purchased from Thermo Fisher Scientific (Waltham, MA). Other reagents and antibodies were the same as described in the previous papers.^[^ ^53^, ^54^ ^]^

### Cell Lines and Cell Culture

All cell lines used in this study were described previously.^[^ ^18^, ^30^, ^55^ ^]^ The cell lines that stably overexpress *TRPM2* gene were established by the infection of lentiviruses carrying a human *TRPM2* gene followed by hygromycin selection. pLenti‐GIII‐CMV (#LV587) and matched vector pLenti‐GIII‐CMV‐TRPM2 (#48 521 061) were purchased from abm (Ferndale, WA). These cell lines have not been genetically authenticated. All cell lines were cultured in RPMI1640 medium supplemented with 5% FBS in a fully humidified incubator, set at 37 °C and 5% CO_2_.

### Colony Formation Assay

PC‐9/AR and HCC827/AR were seeded in 12‐well plates with 200 cells per well. The drugs tested in this study were added after 24 h. The medium was replaced with fresh medium containing the drugs every 3 days. After incubation for 10 days, the medium was removed. The cells were then fixed and stained with 2% crystal violet in ethanol for colony counting and photographed.

### Cell Survival Assay

Cells seeded in 96‐well plates at appropriate densities (3‐6000 cells per well) one day before treatment received drug treatments either alone or in combination for 3 days. Cell numbers were determined by sulforhodamine B (SRB) assay as previously described.^[^ ^56^ ^]^ CI for drug interaction was calculated with the CompuSyn software (ComboSyn, Inc; Paramus, NJ).

### Apoptosis Assays

Detection of apoptosis was done with the annexin V/7‐AAD apoptosis detection kit (BD Biosciences; San Jose, CA) according to the manufacturer's instructions. Apoptosis was also demonstrated by protein cleavages detected with Western blotting.

### Western Blot Analysis

Cells were washed in PBS 3 times and then lysed in lysis buffer (50 mM Tris‐HCl (pH 8.0), 150 mM NaCl, 0.1% SDS, 1% nonidet P‐40, 1 mM phenylmethylsulfonyl fluoride, 5 µg mL^−1^ aprotinin and 5 µg mL^−1^ leupeptin). After the whole‐cell protein lysates were prepared, immunoblotting was done as described previously.^[^ ^53^ ^]^ Protein band intensities were quantified by NIH ImageJ software.

### RT‐qPCR and RNA‐Seq

Cellular total RNA was extracted using an RNA extraction kit (Qiagen; Germantown, MD) according to the manufacturer's instructions. The NanoDrop (Thermo Fisher Scientific) was utilized to measure RNA concentrations. Reverse transcription was completed using the RevertAid First Strand cDNA Synthesis Kit (Qiagen). qPCRs were performed as follows: 95 °C for 15 s followed by 40 cycles of 95 °C for 5 s and 60 °C for 30 s using the QuantStudio 3 and 5 systems (Thermo Fisher Scientific). The primer pairs for TRPM2 were 5′‐ GGCAGCCTTGTACTTCAGTGAC −3′ (forward) and 5′‐ GAGGCAGAACAGGATGAAGTCC −3′ (reverse); GAPDH was used as an endogenous control and detected with the primers of 5′‐GTCTCCTCTGACTTCAACAGCG‐3′ (forward) and 5′‐ACCACCCTGTTGCTGTAGCCAA‐3′. mRNA expression was also detected with RNA‐seq analysis by MedGenome Inc. (Foster City, CA). Differential expression analysis was performed using DESeq2. The expression values for each gene were presented in FPKM (fragments per kilobase per million) units.

### Ca^2+^ Influx Assay

Cells seeded on confocal dishes were exposed to the tested agents for a given time and then loaded with 1 µM Fluo‐4/AM (F14201, Invitrogen) in HBSS (Gibco) for 30 min at 37 °C in the dark. H_2_O_2_ (3 mM) was then added to activate TRPM2 to elicit extracellular Ca^2+^ entry. The cells were bathed in 2.5 mM Ca^2+^‐HBSS that contained the following components in mM: 140 NaCl, 5 KCl, 2.5 CaCl_2_, 0.4 MgSO_4_, 0.5 MgCl_2_, 4 NaHCO_3_, 0.3 NaHPO_4_, 0.4 KH_2_PO_4_, 6 glucose, pH 7.4. Fluo‐4 was excited at 488 nm and captured at wavelengths of 505–530 nm. Data acquisition was performed using the Leica TCS SP8 confocal microscope system. The amplitude of Ca^2+^ response was displayed as a ratio of fluorescence (or maximal fluorescence) relative to the intensity before the application of H_2_O_2_ (F1/F0 or Fmax/F0).

### ROS Detection

Cells seeded on confocal dishes and treated with the tested agents for a given time were washed with HBSS (Gibco) 3 times. The cells were then incubated with 5 µM H_2_DCFDA (D339, Invitrogen) for 45 min at 37 °C in the dark. After removing the loading buffer, cells were washed with HBSS 3 times. Then, a confocal microscope (Leica TCS SP8) was used to collect the images and acquire the data.

### Oligonucleotide Pulldown Assay

The biotin‐linked oligonucleotides were synthesized by Eurofins Genomics (Louisville, KY). The sequences for oligonucleotides with WT VDRE were biotin‐5′‐TGGTGGATCGCCTGAGGTCAGGAGTTTGAGACCAACCTG‐3′ (VDRE‐wt, forward) and 5′‐CAGGTTGGTCTCAAACTCCTGACCTCAGGCGATCCACCA‐3′‐biotin (VDRE‐wt, reverse). The sequences for oligonucleotides with mutated VDRE were biotin‐5′‐TGGTGGATCGCCTGTTCGTCGGAACCCACGACCAACCTG‐3′ (VDRE‐m, forward) and 5′‐CAGGTTGGTCGTGGGTTCCGACGAACAGGCGATCCACCA‐3′‐biotin (VDRE‐m, reverse). The sequences for the irrelevant scramble oligonucleotides were biotin‐5′‐ACTGACTGACTGACTGACTGACTG‐3′ (scramble, forward), and 5′‐CAGTCAGTCAGTCAGTCAGTCAGT‐3′ (scramble, reverse). Double‐stranded oligonucleotides were annealed following a standard protocol. The oligonucleotide pulldown assay was carried out as described previously.^[^ ^57^ ^]^ In brief, the biotinylated double‐stranded oligonucleotides and ImmunoPure streptavidin‐agarose beads (Thermo Fisher Scientific/Pierce, Waltham, MA) were mixed with 200 µg of whole‐cell protein lysate followed by incubation at 4 °C with shaking for 16 h. The beads were then pelleted and washed with cold lysis buffer for 4 times. Bound proteins were finally separated by SDS‐PAGE followed by Western blot analysis to detect protein of interest with a specific antibody.

### Transient Transfection and Luciferase Reporter Assay

Cells in 24‐well plates were transfected with a TRPM2 reporter plasmid together with a pCH110 plasmid harboring a β‐galactosidase gene using Fugene 6 reagent (Roche Applied Science, Indianapolis, IN) following the manufacturer's protocol. After 24 h, the cells were treated with DMSO or Osim in serum‐free medium for 16 h and then harvested in cell‐lysis buffer. Luciferase activity was measured with the Luciferase Assay kit (Promega; Madison, WI) using a Sirius Luminometer (Berthold Detection Systems; Huntsville, AL). The luciferase activity was normalized with the β‐galactosidase activity, which was measured as described previously.^[^ ^58^ ^]^

### Gene Knockdown using siRNA and shRNA

*TRPM2* siRNA (sc‐42674), c‐*Myc* siRNA (sc‐29226), *VDR* siRNA (sc‐106692), and *VDR* shRNA plasmid (sc‐106692‐SH) were purchased from Santa Cruz Biotechnology. *TRPM2* shRNAs including shTRPM2#1 (TRCN0000044148), shTRPM2#2 (TRCN0000044150), shTRPM2#3 (TRCN0000150664), shTRPM2#4 (TRCN0000154454) and shTRPM2#5 (TRCN0000157623) were purchased from MilliporeSigma. Scramble control and the procedures used for transfection were described previously.^[^ ^54^ ^]^

### Detection of DTCs

Cells seeded in 12‐well plates at a density of almost 90% were exposed to the tested drugs. The medium was replaced with fresh medium containing the same drugs every 2 days. After incubation for 5 or 10 days, the medium was removed for fixing and staining DTCs with 2% crystal violet in ethanol.

### TCGA Data Analysis

Kaplan‐Meier analysis was performed using EGFR TKI–treated patient data retrieved from TCGA LUAD datasets (http://cancergenome.nih.gov/). The patients were stratified according to high versus low expression (cutoff: median) of *TRPM2* or *VDR* within their tumors.

### Human NSCLC Tissues

Paired tissue samples from patients with EGFRm NSCLC before treatment (i.e., baseline) and after disease relapse from treatment with EGFR‐TKIs such as gefitinib or osimertinib, were collected at the Second Xiangya Hospital (Changsha, Hunan, China) and Henan Cancer Hospital (Zhengzhou, Henan, China) under Ethics Review Committee (IRB)‐approved protocols (Xiangya IRB2019‐009, Henan IRB2019‐067 and Emory IRB00104138). All tissues were sent to and stained at the Second Xiangya Hospital.

### IF Staining

Tissue slides or cells grown on chamber slides were fixed with 4% paraformaldehyde for 10 min, then washed with PBS 3 times and incubated with blocking buffer (5% BSA containing with 0.2% Triton X‐100) at room temperature for 30 min. Then slides were exposed to primary antibodies (anti‐TRPM2 1:100, anti‐VDR 1:100, or anti‐p‐H2AX 1:100) overnight at 4 °C, then incubated with the secondary antibodies Alexa Fluor 488‐Donkey anti‐mouse (1:200) or Alexa Fluor 568‐Donkey anti‐rabbit (1:200) for 1 h at room temperature. Slides were mounted in medium containing DAPI. Images were collected using confocal microscopy (Leica TCS SP8).

### Immunohistochemistry (IHC)

Tissue slides were dewaxed with xylene, followed by rehydration with a graded alcohol series. Tissue slides were exposed to 3% (v/v) hydrogen peroxide for 10 min and then incubated with blocking buffer (10% BSA in PBS) for 1 h. After blocking, the slides were incubated with primary antibodies (Ki‐67 1:100 and cleaved PARP 1:50) at 4 °C in a humidified chamber overnight and then treated with ImmPRESS Horse Anti‐Rabbit IgG Polymer Kit as described in our previous study.^[^ ^54^ ^]^ Human NSCLC tissues were stained using the EnVision + Dual Link System‐HRP Kit (Dako; Carpinteria, CA) as described previously.^[^ ^59^ ^]^ TRPM2 antibody (#ACC‐043; Alomone Labs) was diluted at 1:1200.

### Animal Xenograft and Treatments

Animal experiments were approved by the Institutional Animal Care and Use Committee (IACUC) of Emory University (PROTO201700718). In the conventional cell‐derived xenograft studies, cells suspended in sterile PBS at 3 × 10^6^ per mouse were injected into the flank of 4‐week‐old nu/nu nude mice purchased from The Jackson Laboratory (Bar Harbor, Maine). On day 7, when the average tumor was ≈80 mm^3^, the mice were divided into groups with equal average tumor volumes and body weights. The following treatments were administered daily: vehicle, osimertinib (5 mg kg^−1^, og), D9 (25 mg kg^−1^, ip) or ACA (15 mg kg^−1^, ip), and the combination of osimertinib and D9 or combination of osimertinib and ACA. Tumor volume was measured using calipers every 2 or 3 days and calculated by V = π (length x width2)/6. Body weight was also measured every 2 or 3 days. At the end of the experiment, mice were sacrificed using CO_2_. The tumors were then removed, weighed, and stored in formalin for further analysis.

In PDX studies, the three PDXs harboring different EGFR mutations, TM00193 (E746_A750del), TM00199 (L858R), and TM00219 (E746_A750del; T790M; exon 19 del), were purchased from The Jackson Laboratory. When the average tumor was around 100 mm^3^, the mice were treated with vehicle, osimertinib (5 mg kg^−1^, og), D9 (25 mg kg^−1^, ip), and the combination of osimertinib with D9 every day. Tumor volume was measured using calipers every 3 or 4 days.

For the above animal experiments, vehicle and osimertinib treatment groups were shared with other treatments as described in a previous study^[^ ^60^ ^]^ to minimize utilization of mice.

### Statistical Analysis

Statistical differences between two groups and among multiple groups were determined by two‐sided unpaired or paired Student's *t*‐test and one‐way ANOVA test, respectively. Results are presented as means ± SDs or SEs. All statistical analyses were conducted using Graphpad Prism 9.0 software. P values less than 0.05 were considered statistically significant.

## Conflict of Interest

SSR is on consulting/advisory board for AstraZeneca, BMS, Merck, Roche, Tesaro and Amgen.

## Supporting information

Supporting Information

Supplemental Data 1

## Data Availability

The data that support the findings of this study are available from the corresponding author upon reasonable request.
