# Supplementary material for: Targeting Transient Receptor Potential Melastatin‐2 (TRPM2) Enhances Therapeutic Efficacy of Third Generation EGFR Inhibitors against EGFR Mutant Lung Cancer
Source: Adv Sci (Weinh). 2024 Jul 23;11(35):2310126. doi: 10.1002/advs.202310126 (PMC11425210; doi:10.1002/advs.202310126)
Supplement: Supplementary file 1 — Supporting Information [file ADVS-11-2310126-s002.pdf]

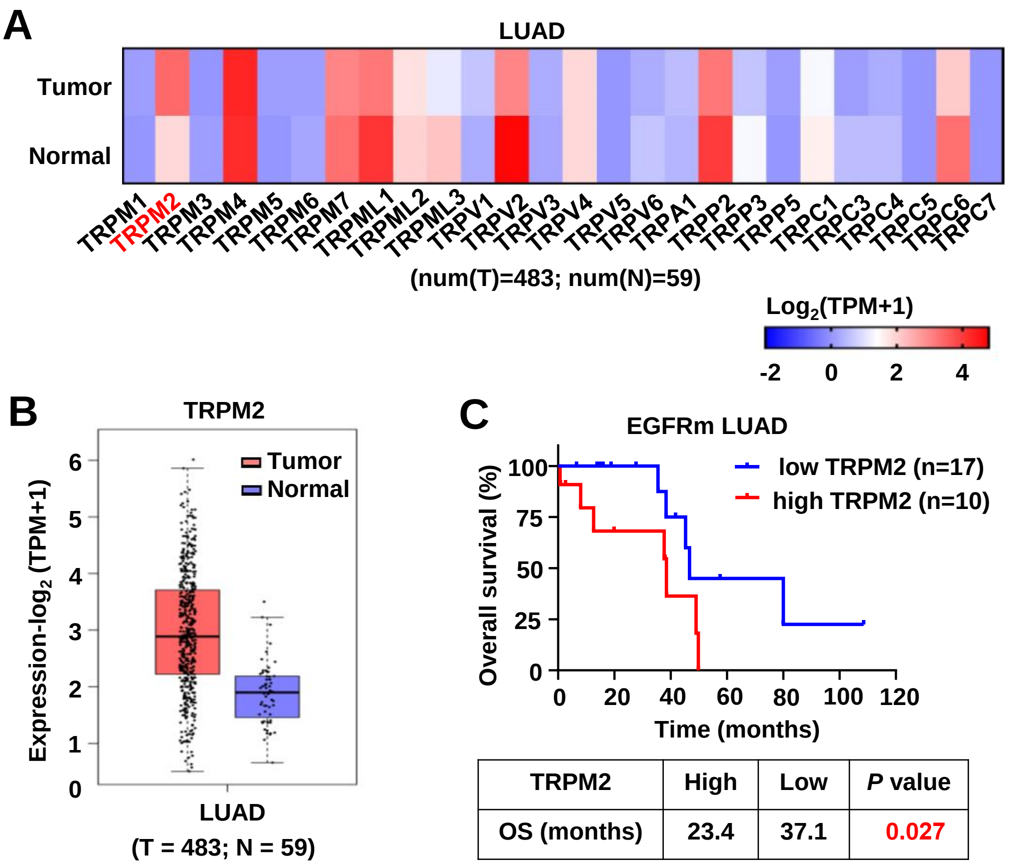

TRPM2	High	Low	*P* value
OS (months)	23.4	37.1	0.027

Figure S1. TCGA data analysis of TRPM2 expression in normal tissues and lung adenocarcinoma (LUAD; *A* and *B*) and the impact of TRPM2 expression on the survival of patients with EGFRm LUAD (*C*).

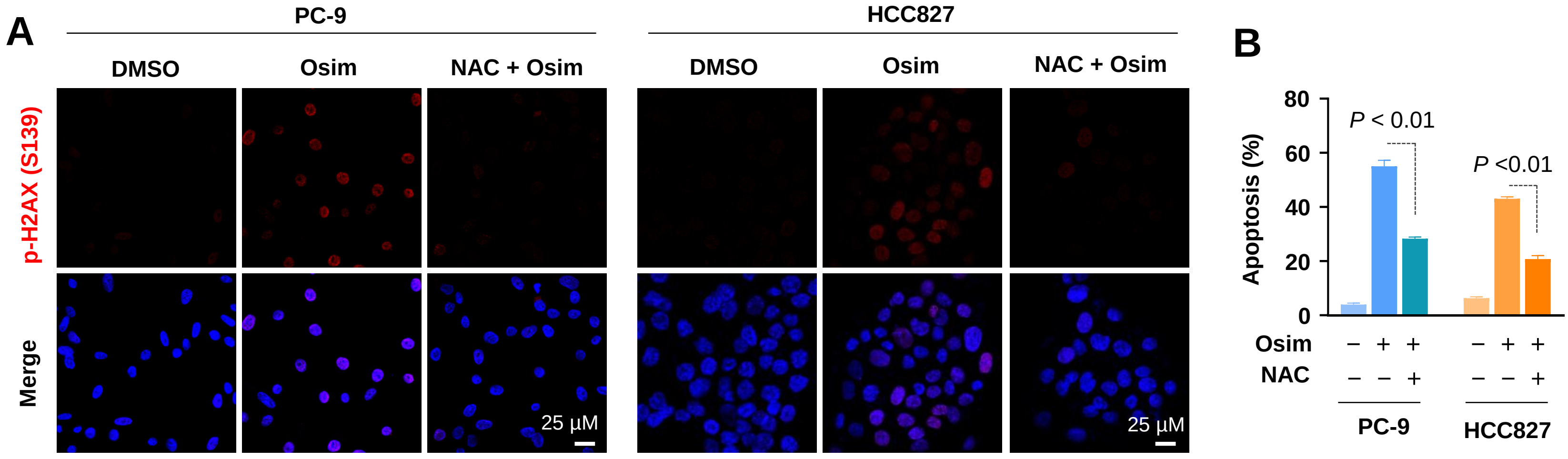

Figure S2. The presence of NAC attenuates induction of DNA damage (*A*) and apoptosis (*B*) by osimertinib in EGFRm NSCLC cells. The indicated cell lines were pre-treated with 3 mM NAC for 30 min and then co-treated with 100 nM osimertinib (Osim) for 24 h (*A*) or 48 h (*B*) and then subject to p-H2AX staining (*A*) and detection of apoptosis using annexin V staining and flow cytometric analysis. The data in *B* are means ± SDs of triplicate determinations. The statistic differences were evaluated with two-sided unpair Student's t test.

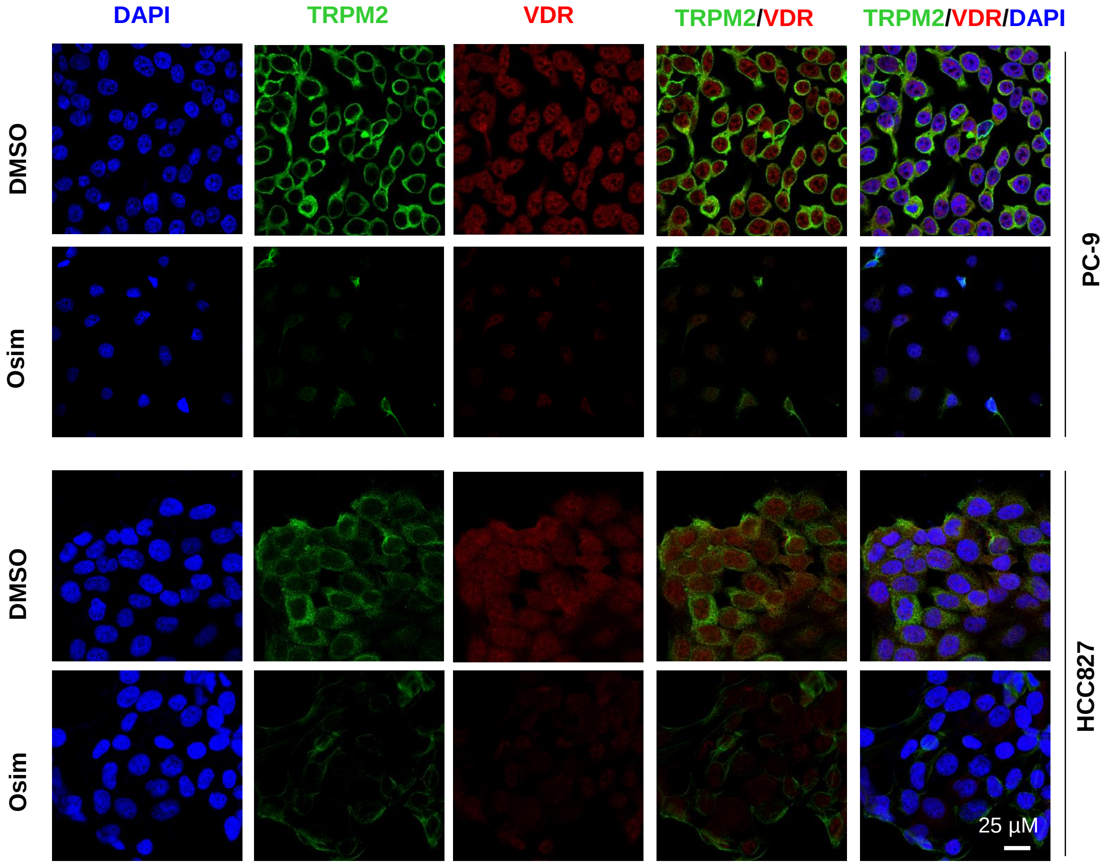

Figure S3. Co-staining of TRPM2 and VDR in EGFRm NSCLC cell lines exposed to osimertinib. After the indicated cell lines were exposed to 100 nM osimertinib (Osim) for 16 h, the cells were then subjected to IF co-stained with TRPM2 and VDR antibodies, respectively.

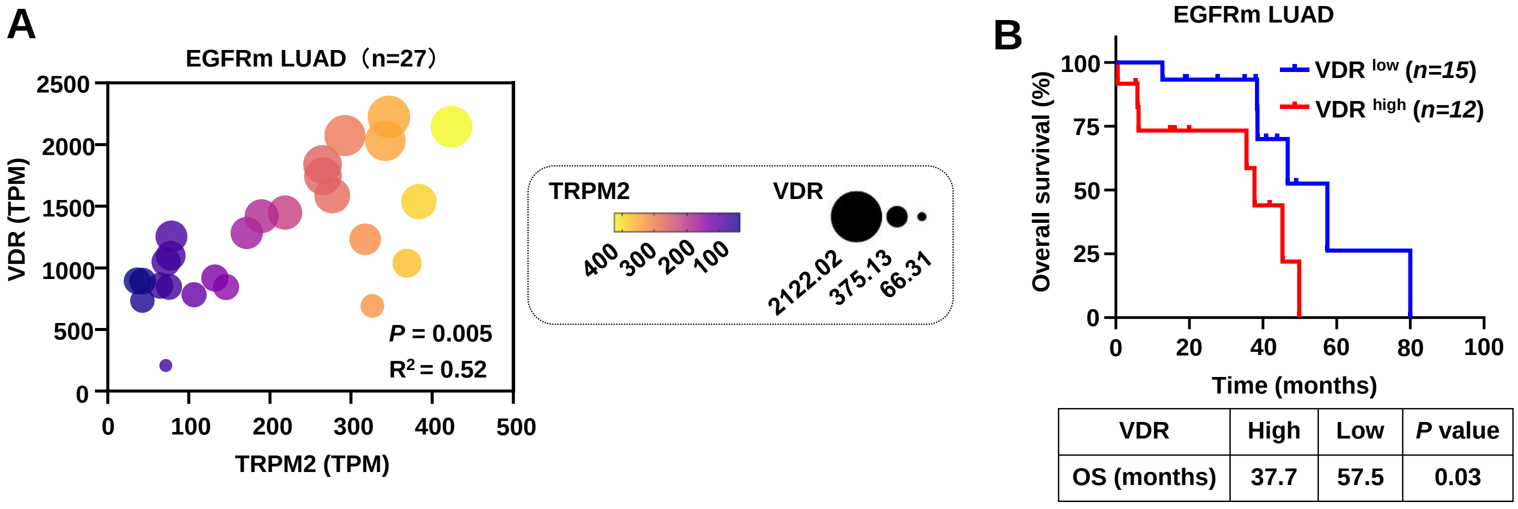

VDR	High	Low	*P* value
OS (months)	37.7	57.5	0.03

Figure S4. TCGA data analysis of the correlation between VDR and TRPM2 expression in EGFRm lung adenocarcinoma (LUAD; *A*) and the prognostic impact of VDR expression in EGFRm LUAD (*B*).

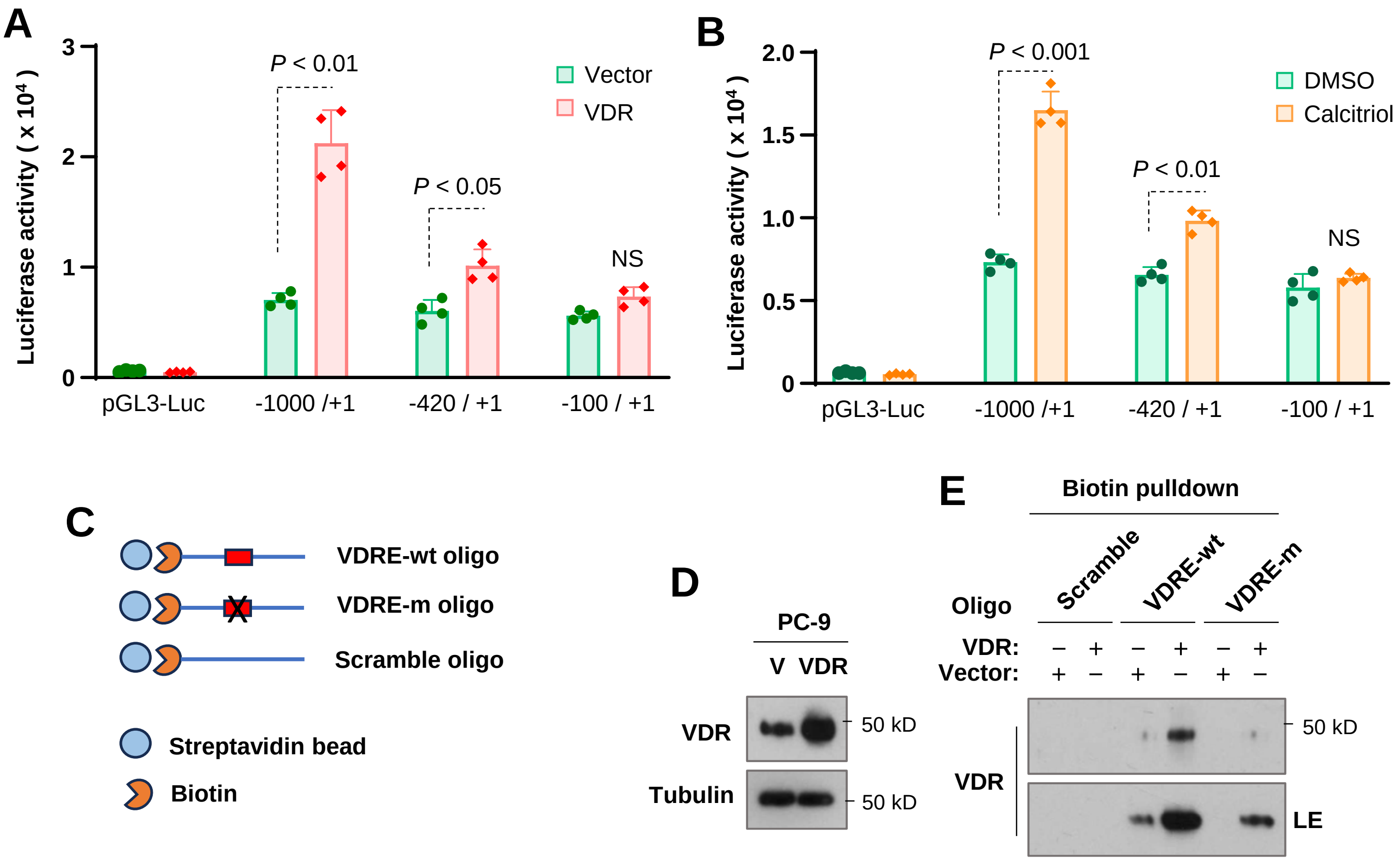

Figure S5. VDR expression (*A*) and calcitriol (*B*) increase TRPM2 transcription primarily in a VDRE-dependent manner (*C*-*E*). *A*, HEK293T cells were co-transfected with the individual reporter constructs as indicated and VDR-expressing plasmid for 24 h. *B*, HEK293T cells were transfected with the indicated reporter constructs for 24 h followed by exposure to 100 nM calcitriol for an additional 16 h. The cells were then harvested and lysed for luciferase activity assay. The data are means ± SDs of four replicate determinations. Statistic differences were evaluated with two-side unpair Student's t test. *C*, Schema for the streptavidin/biotin pulldown assay. *D* and *E*, PC-9 cells were transfected with vector (V) or VDR expression plasmid for 48 h and then collected for preparation of whole-cell protein lysates and subsequent Western blotting (*D*) and biotin pulldown/Western blotting (*E*) to detect VDR. LE, long exposure.

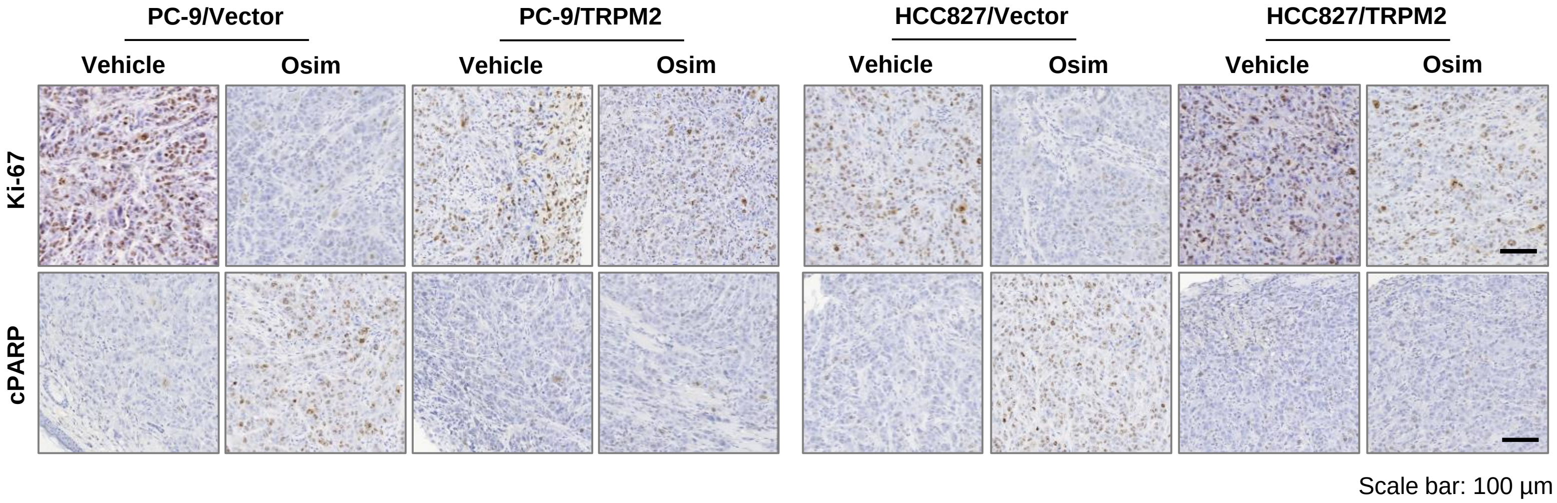

Figure. S6. IHC detection of Ki-67 and cPARP in tumor tissues treated with osimertinib. The tumor treatments were the same as described in Fig. 3.

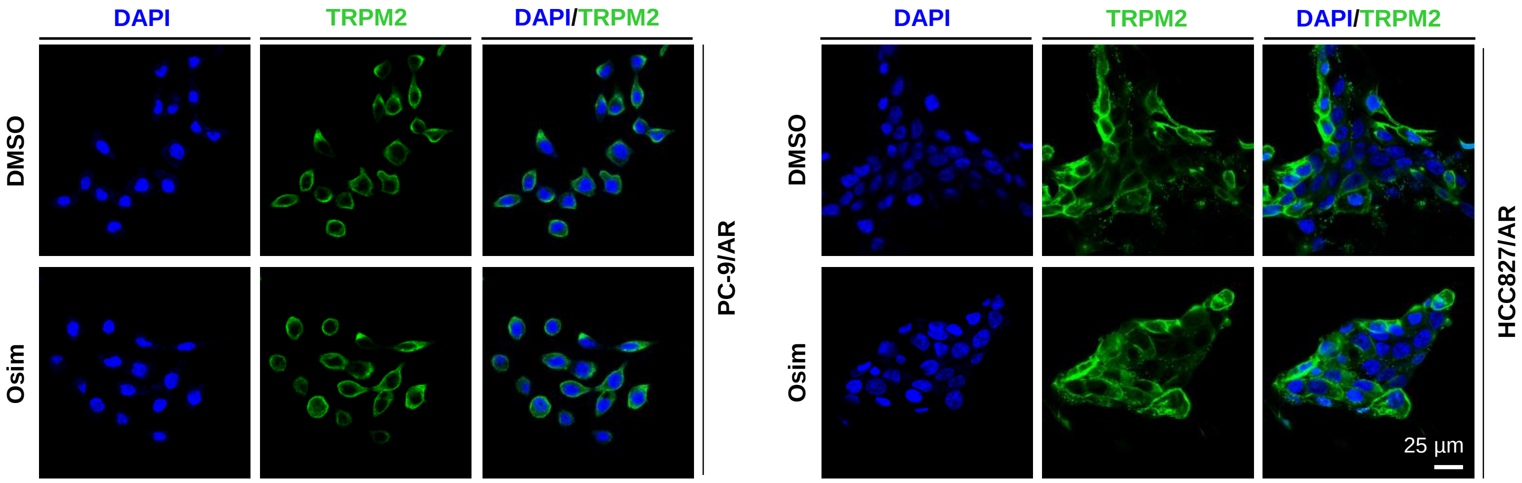

Figure S7. IF staining of TRPM2 in EGFRm NSCLC cell lines with acquired resistance to osimertinib. The indicated cell lines were exposed to 100 nM osimertinib (Osim) for 24 h before the IF was conducted.

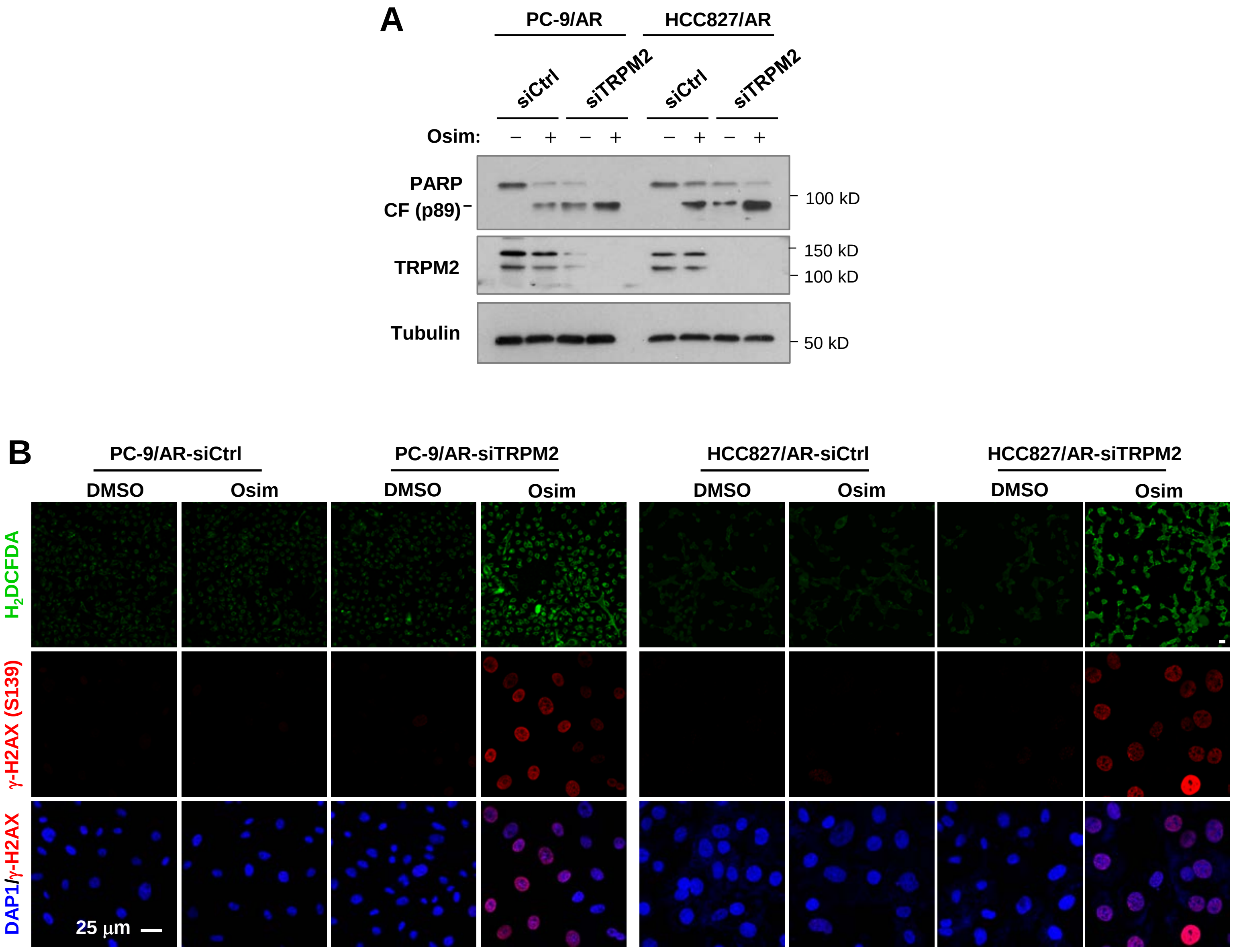

Figure S8. Effects of siRNA-mediated TRPM2 knockdown in osimertinib-resistant EGFRm NSCLC cell lines on induction of apoptosis (*A*), ROS generation and DNA damage (*B*) by osimertinib. The indicated cell lines were transfected with control (Ctrl) or TRPM2 siRNA and after 48 h were treated with DMSO or 100 nM osimertinib (Osim) for an additional 24 h (*A*) or 16 h (*B*). The proteins of interest were detected with Western blotting (*A*). ROS generation and DNA damage were detected with H2DCFDA and γ-H2AX foci assays, respectively. CF, cleaved form.

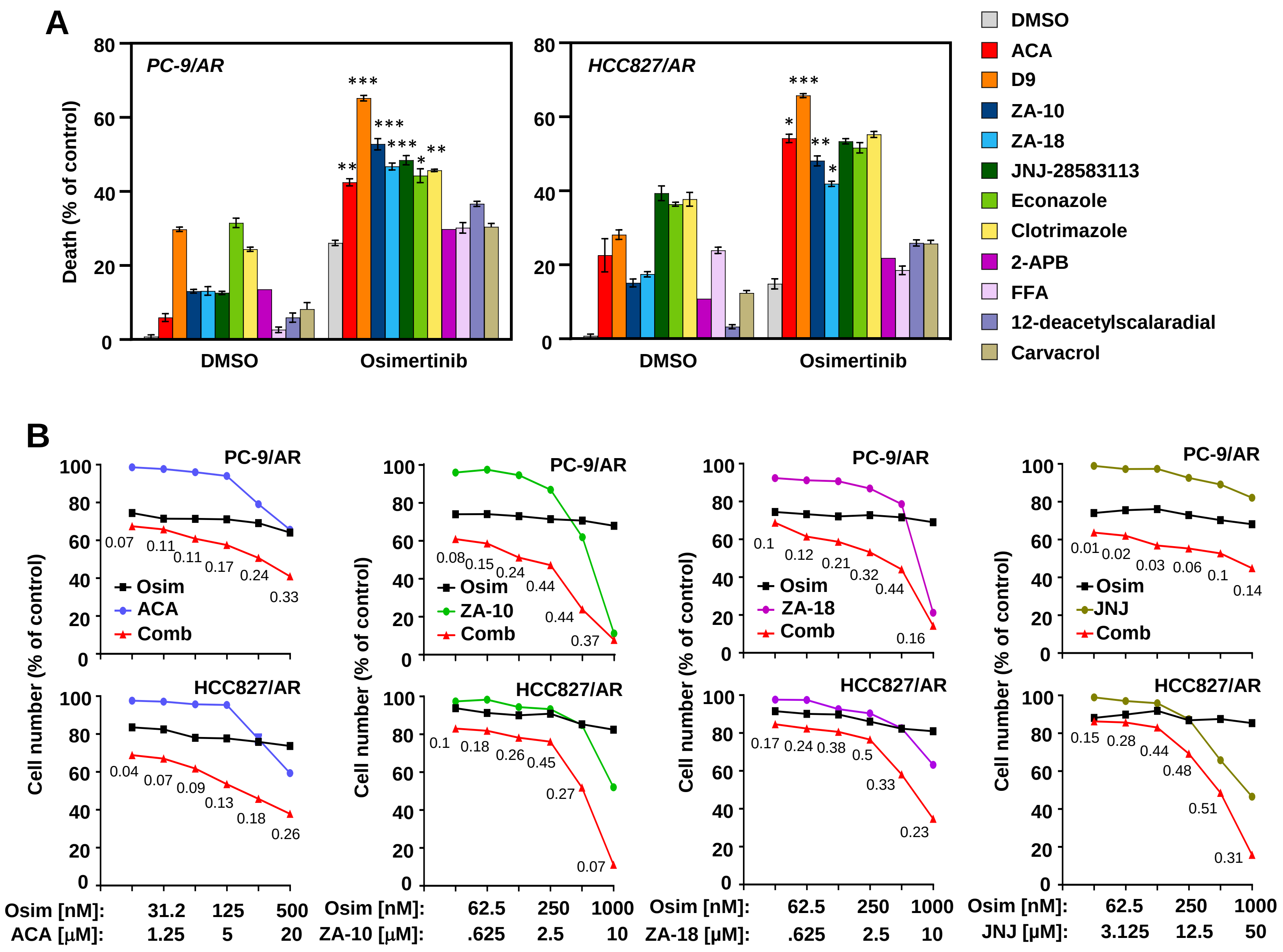

Figure S9. Effects of osimertinib combined with different TRPM2 inhibitors on the survival of EGFRm NSCLC cell lines with acquired resistance to osimertinib. *A*, The indicated cell lines were treated with 200 nM osimertinib alone, the tested agent alone or their respectively combinations for 3 days. The concentrations of ACA, D9, JNJ-28583113, econazone, clotrimazole, 2-APB, FFA, 12-decetylscalarradial and carvacrol were 10 µM. The concentrations of ZA-10 and ZA-18 were 5 µM. *B*, The given cell lines were treated with the varied concentrations of osimertinib alone, the tested TRPM2 inhibitor alone or their respective combinations for 3 days. Cell numbers were estimated with the SRB assay. The numbers inside the graphs by the red lines are CIs for the indicated combinations. The data are means + SD of four replicate determinations. JNJ, JNJ-28583113. *, P < 0.05; **, P < 0.01; and ***. P < 0.001 compared with the effect of each agent alone using one way ANOVA test.

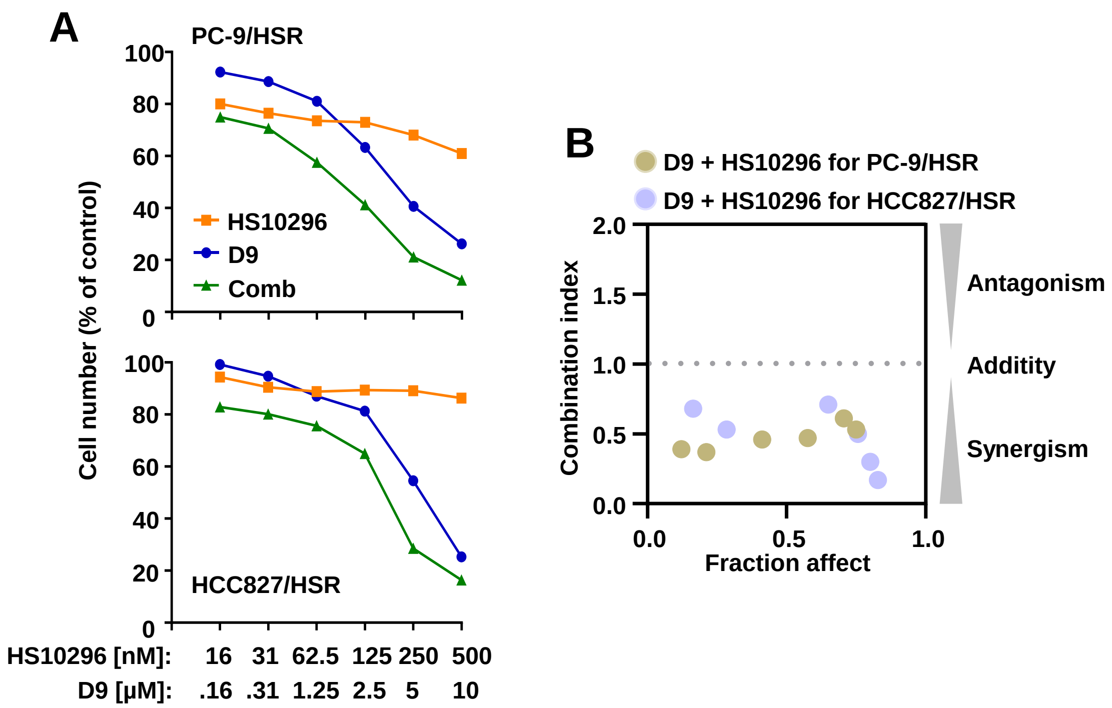

Figure S10. Synergistic effects of D9 combined with HS10296 on the survival of EGFRm NSCLC with acquired resistance to HS10296. The tested cell lines were exposed to varied concentrations of D9 alone, HS10296 alone or their combination for 3 days. Cell numbers were estimated with the SRA assay. The data are means ± SDs of four replicate determinations (*A*). CIs were also calculated and presented (*B*).

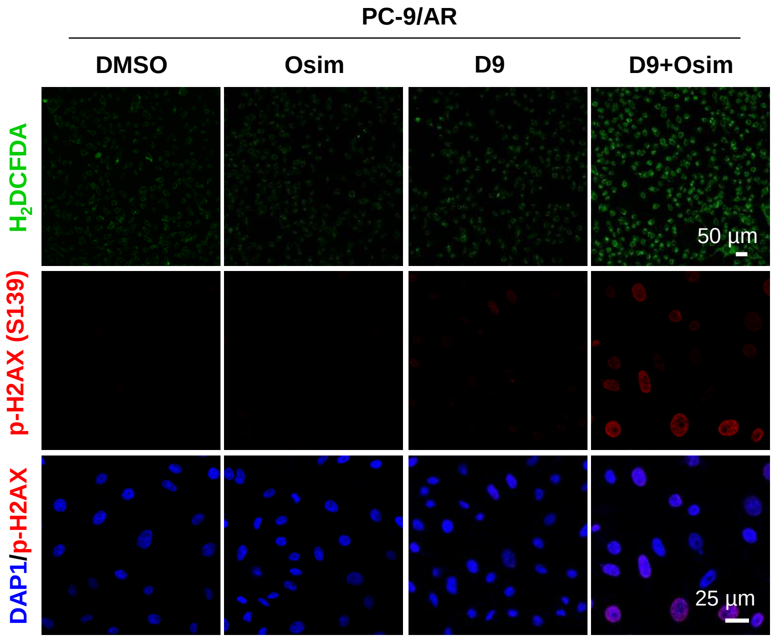

Figure S11. The combination of osimertinib and D9 enhances ROS generation assayed with H_2DCFDA and DNA damage detected with p-H2AX staining in PC-9 AR cells. Cell treatments were the same as described in Fig. 6F.

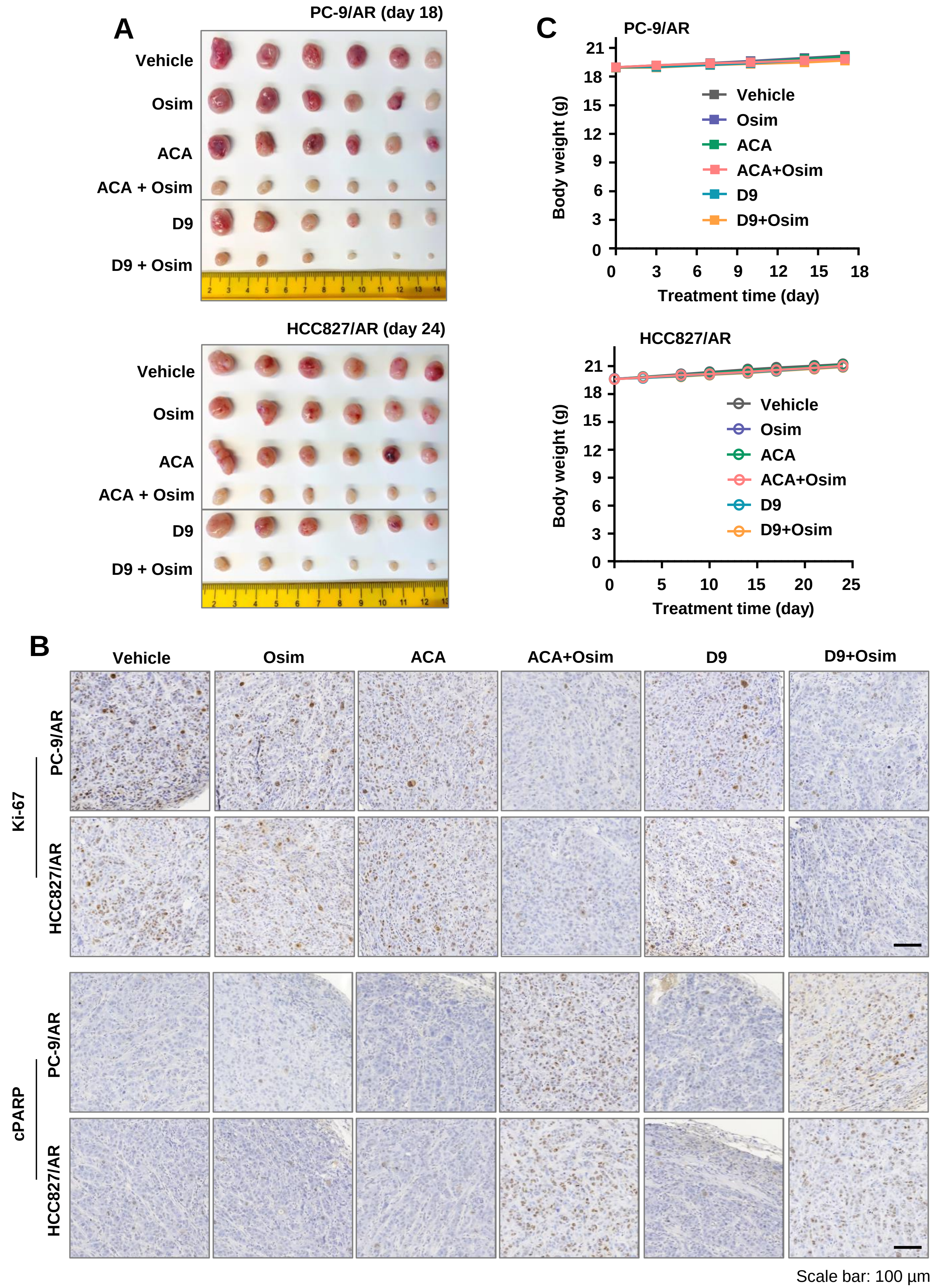

Figure S12. Tumor sizes (*A*), IHC detection of Ki-67 and cPARP in tumor tissues (*B*) and mouse body weight alterations (*C*). Mouse treatments were the same as described in Fig. 6.

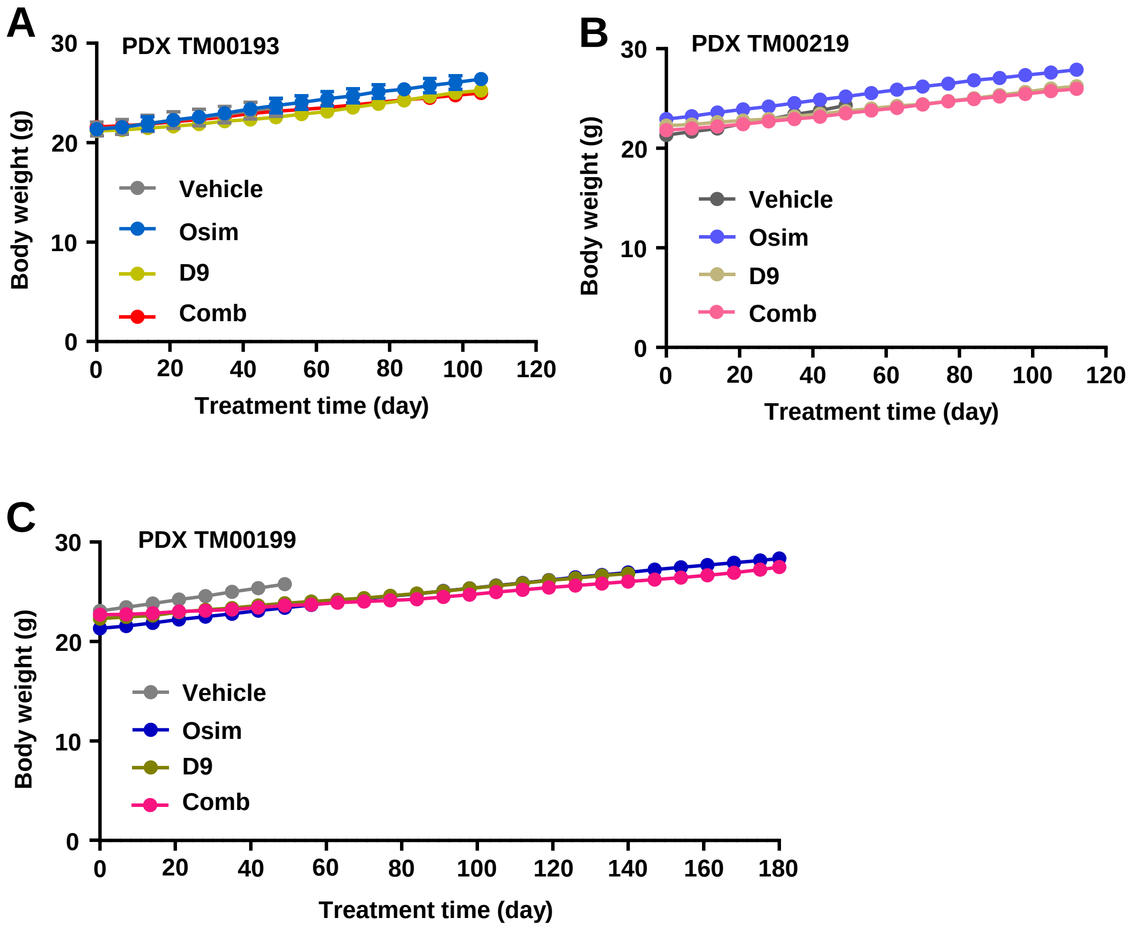

Figure S13. The combination of osimertinib with D9 does not show enhanced effects on reducing mouse body weights. The treatment of mice was the same as described in Figure 7.
